# Constraints on the Cycling of Iron Isotopes From a Global Ocean Model

**DOI:** 10.1029/2021GB006968

**Published:** 2021-09-16

**Authors:** D. König, T. M. Conway, M. J. Ellwood, W. B. Homoky, A. Tagliabue

**Affiliations:** ^1^ School of Environmental Sciences University of Liverpool Liverpool UK; ^2^ College of Marine Science University of South Florida St Petersburg FL USA; ^3^ Research School of Earth Sciences Australian National University Canberra ACT Australia; ^4^ School of Earth and Environment University of Leeds Leeds UK

**Keywords:** biogeochemistry, iron isotopes, ocean, model

## Abstract

Although iron (Fe) is a key regulator of primary production over much of the ocean, many components of the marine iron cycle are poorly constrained, which undermines our understanding of climate change impacts. In recent years, a growing number of studies (often part of GEOTRACES) have used Fe isotopic signatures (δ^56^Fe) to disentangle different aspects of the marine Fe cycle. Characteristic δ^56^Fe endmembers of external sources and assumed isotopic fractionation during biological Fe uptake or recycling have been used to estimate relative source contributions and investigate internal transformations, respectively. However, different external sources and fractionation processes often overlap and act simultaneously, complicating the interpretation of oceanic Fe isotope observations. Here we investigate the driving forces behind the marine dissolved Fe isotopic signature (δ^56^Fe_diss_) distribution by incorporating Fe isotopes into the global ocean biogeochemical model PISCES. We find that distinct external source endmembers acting alongside fractionation during organic complexation and phytoplankton uptake are required to reproduce δ^56^Fe_diss_ observations along GEOTRACES transects. δ^56^Fe_diss_ distributions through the water column result from regional imbalances of remineralization and abiotic removal processes. They modify δ^56^Fe_diss_ directly and transfer surface ocean signals to the interior with opposing effects. Although attributing crustal compositions to sedimentary Fe sources in regions with low organic carbon fluxes improves our isotope model, δ^56^Fe_diss_ signals from hydrothermal or sediment sources cannot be reproduced accurately by simply adjusting δ^56^Fe endmember values. This highlights that additional processes must govern the exchange and/or speciation of Fe supplied by these sources to the ocean.

## Key Points

- External source endmembers and fractionation during phytoplankton uptake and organic complexation shape oceanic Fe isotope distribution
- Imbalances in remineralization and abiotic removal rates produce regionally distinct dissolved Fe isotopic signatures
- Hydrothermal and sedimentary Fe supply processes have additional controls that are not yet accounted for in global ocean models

## 1 Introduction

Iron (Fe) is a key micronutrient in the marine environment, directly limiting phytoplankton growth and net primary production over much of the global surface ocean, and also an important control on nitrogen fixation. Fe availability is thus a key factor in modulating the ocean carbon cycle over multiple timescales (Moore et al., 2013; Tagliabue et al., 2017). Despite this broad understanding of the role of Fe in marine biogeochemical cycles, many important uncertainties remain, largely due to the array of overlapping processes in operation (Tagliabue et al., 2017). For example, while atmospheric dust deposition, sediments, and hydrothermal vents are all now recognized as major external Fe sources, the extent of their respective input fluxes remains poorly constrained (Boyd & Ellwood, 2010; Homoky et al., 2016; Tagliabue et al., 2016). Inorganic Fe is poorly soluble in seawater and readily removed, but is stabilized in the dissolved phase (dFe) via complexation with organic moieties of various origin, binding strength, and often poorly defined molecular structure (referred to as “ligands” hereafter), which therefore regulate its bioavailability and oceanic residence time (Gledhill & Buck, 2012; Tagliabue, Aumont, et al., 2014). Fe uptake by phytoplankton and bacteria alongside abiotic processes such as scavenging and “colloidal pumping” depletes dFe. Fe is also heavily recycled between dissolved and biological pools, especially where the input of new Fe is scarce (Boyd et al., 2012; Strzepek et al., 2005; Tagliabue, Sallée, et al., 2014).

The combination of complex internal cycling and poorly defined external supply causes substantial uncertainty in model representations of the marine Fe cycle. For instance, while sediments and hydrothermal vents are thought to regulate primary production in areas with little surface dust Fe input (Boyd & Ellwood, 2010; Tagliabue et al., 2017), poor knowledge of both the speciation of the Fe added and the stabilization mechanism that keeps it in solution lead to divergence in their representation in models (Tagliabue et al., 2016). For example, for the sediment source, models only reflect a reductive input of Fe(II), and scale the magnitude of Fe input, either by water depth (e.g., Aumont et al., 2015) or by the organic carbon flux to sediments (e.g., Moore & Braucher, 2008). Some models add modulation of this input by bottom water oxygen levels (e.g., Dale et al., 2015; Galbraith et al., 2010). Both approaches draw on empirical relationships to derive estimates of how carbon oxidation in sediments modulates Fe fluxes from Fe‐reducing sediments (Elrod et al., 2004; Middelburg et al., 1997), and ignore the evidence for the release of dFe from oxic sediments (Homoky et al., 2013, 2021). For hydrothermal vents, despite noted local inter‐site variations (Tagliabue et al., 2010), Fe inputs are typically tied to ^3^He supply via a simple fixed dFe/^3^He ratio (Resing et al., 2015; Roshan et al., 2020; Tagliabue et al., 2010). Including the stabilization of hydrothermal Fe in models has been shown to be important in reproducing Fe observations around some ridges, but not all (e.g., Resing et al., 2015; Tagliabue & Resing, 2016). Thus, a wide variety of Fe cycle parameterizations are used in ocean models (Dale et al., 2015; Roshan et al., 2020; Tagliabue et al., 2016), which ultimately lead to large uncertainty in the role played by Fe in shaping ocean biogeochemical cycles.

As with the macronutrients (e.g., nitrogen; Sigman & Fripiat, 2019), additional insight into the cycling of dFe may be gained from its isotope signatures (mostly δ^56^Fe), which are a passive tracer and have been used to examine both dFe sources (e.g., Conway & John, 2014; Radic et al., 2011), and internal cycling (e.g., Ellwood et al., 2015, 2020; Sieber et al., 2021). However, this requires disentanglement of both the role of primary source δ^56^Fe endmembers and the impact of a range of internal cycling processes. For dust or oxic sediment sources, dFe is supplied with δ^56^Fe endmembers close to crustal values (+0.09‰; Beard et al., 2003; Conway et al., 2019; Homoky et al., 2013; Radic et al., 2011; Waeles et al., 2007). More variable δ^56^Fe endmembers are typical in source systems where redox conditions are more variable, such as oxygen‐depleted sediments or hydrothermal vents, due to the large fractionation between light Fe(II) and heavy Fe(III) at equilibrium (Welch et al., 2003). This leads to increased light δ^56^Fe that range from around −0.5‰ to −0.1‰ for hydrothermal vent fluids (Johnson et al., 2020 and references therein) and as low as −3.3‰ for Fe^2+^ produced by microbial dissimilatory reduction in sediment porewaters (e.g., Homoky et al., 2009; Severmann et al., 2010). The “true” source δ^56^Fe endmembers of hydrothermal or reducing sediment settings may also be altered as Fe(II) is oxidized, then precipitated or complexed, in the predominantly oxic water column, complicating efforts to characterize the net effect on δ^56^Fe_diss_ of these Fe sources (e.g., Chever et al., 2015; Lough et al., 2017). Indeed, dFe complexation by ligands is thought to preferentially bind heavy Fe, whereby the extent of such fractionation may depend on ligand type or binding strength (Dideriksen et al., 2008; Morgan et al., 2010). This process has also been invoked to explain why the net δ^56^Fe_diss_ of dust‐derived and hydrothermally derived dFe may ultimately be heavier than its source (e.g., Conway & John, 2014; Fitzsimmons et al., 2017).

In addition to variable source δ^56^Fe endmembers, the open system nature of the global ocean and internal fractionation processes may also impact δ^56^Fe_diss_ distributions. For instance, the removal of light particulate Fe (pFe) by sinking was found to be a key process behind the increasing δ^56^Fe_diss_ observed in a Southern Ocean eddy (Ellwood et al., 2020). The impact of biological cycling on δ^56^Fe_diss_ is also likely to be complex, with phytoplankton inferred to preferentially take up light dFe in some studies (Ellwood et al., 2015, 2020; Radic et al., 2011; Sieber et al., 2021), or with no or opposite effects in other studies (Conway & John, 2014; Klar et al., 2018). Release of light dFe during remineralization of pFe has been proposed from field observations (Abadie et al., 2017; Ellwood et al., 2020), but its extent is unclear, and release of heavy remineralized Fe has been noted elsewhere (Klar et al., 2018). All of this adds complexity to our understanding of field data. Mechanistic ocean biogeochemical models are a useful tool for evaluating the roles played by a diverse range of processes on a larger scale, and account for open‐system conditions. Including Fe isotope cycling in a mechanistic sense should help address questions about the competing roles of uptake and remineralization processes and different external input endmembers in driving the large‐scale δ^56^Fe_diss_ distribution. These questions are difficult to answer from field studies alone. This is especially timely given the rapid increase of δ^56^Fe_diss_ data from ocean sections as part of the GEOTRACES program. Such approaches have previously provided useful insight into the cycling of a range of trace metals sampled by GEOTRACES (such as cobalt, copper, manganese, and zinc; Richon & Tagliabue, 2019; Tagliabue et al., 2018; Van Hulten et al., 2017; Vance et al., 2017; Weber et al., 2018), but have largely ignored metal isotopes (except Weber et al., 2018).

In this study, we add Fe isotopes to a state‐of‐the‐art global ocean biogeochemical model to determine how external Fe sources with different δ^56^Fe endmembers interact with isotope fractionation processes during the internal oceanic cycling of Fe. A set of sensitivity experiments, constrained by available δ^56^Fe_diss_ data from the GEOTRACES program, allow us to show that a combination of variable source endmembers and internal fractionation is necessary to best reproduce observations. We demonstrate that the impact of remineralization on δ^56^Fe_diss_ can vary, and, crucially, depends on upper ocean Fe sources and biological cycling, and is often counterbalanced by abiotic removal processes such as scavenging. We also reveal regional to basin‐scale differences in the impact of hydrothermal sources, and show that sediments generally supply isotopically light Fe into the upper ocean but supply Fe with crustal δ^56^Fe endmember deeper in the water column, possibly regulated by organic carbon supply to the seafloor.

## 2 Methods

### 2.1 PISCES Biogeochemical Model

We incorporated our Fe isotope model into the PISCES model (Aumont et al., 2015); a well‐established global ocean biogeochemical model, which is also used to study the effects of climate change as a part of larger earth system modeling frameworks, for example, as part of the IPSL, CNRM and EC‐EARTH models that participated in CMIP6 (Kwiatkowski et al., 2020).

Here, we started from PISCES v2, which includes two types of phytoplankton and zooplankton, two particle size classes, five nutrients (NO_3_, NH_4_, PO_4_, dFe, and Si(OH)_4_), explicit Fe‐binding ligands, the carbonate system (DIC, alkalinity, and CaCO_3_), DOC, O_2_, and accounts for CaCO_3_ production/dissolution, nitrogen fixation, sedimentary and water column denitrification, and anammox. PISCES is embedded in the NEMO ocean model framework and here we use the ORCA2 configuration with a horizontal resolution of 2° by 2° the cosine of the latitude, with the latitudinal resolution enhanced to 0.5° in the equatorial regions. The model has 31 depth levels (bounded at 0, 10, 20, 30, 40, 50, 60, 70, 80, 90, 101, 111, 123, 135, 150, 169, 197, 241, 313, 430, 612, 873, 1,212, 1,613, 2,057, 2,527, 3,012, 3,504, 4,001, 4,500, 5,000, and 5,500 m).

**Figure 1:**
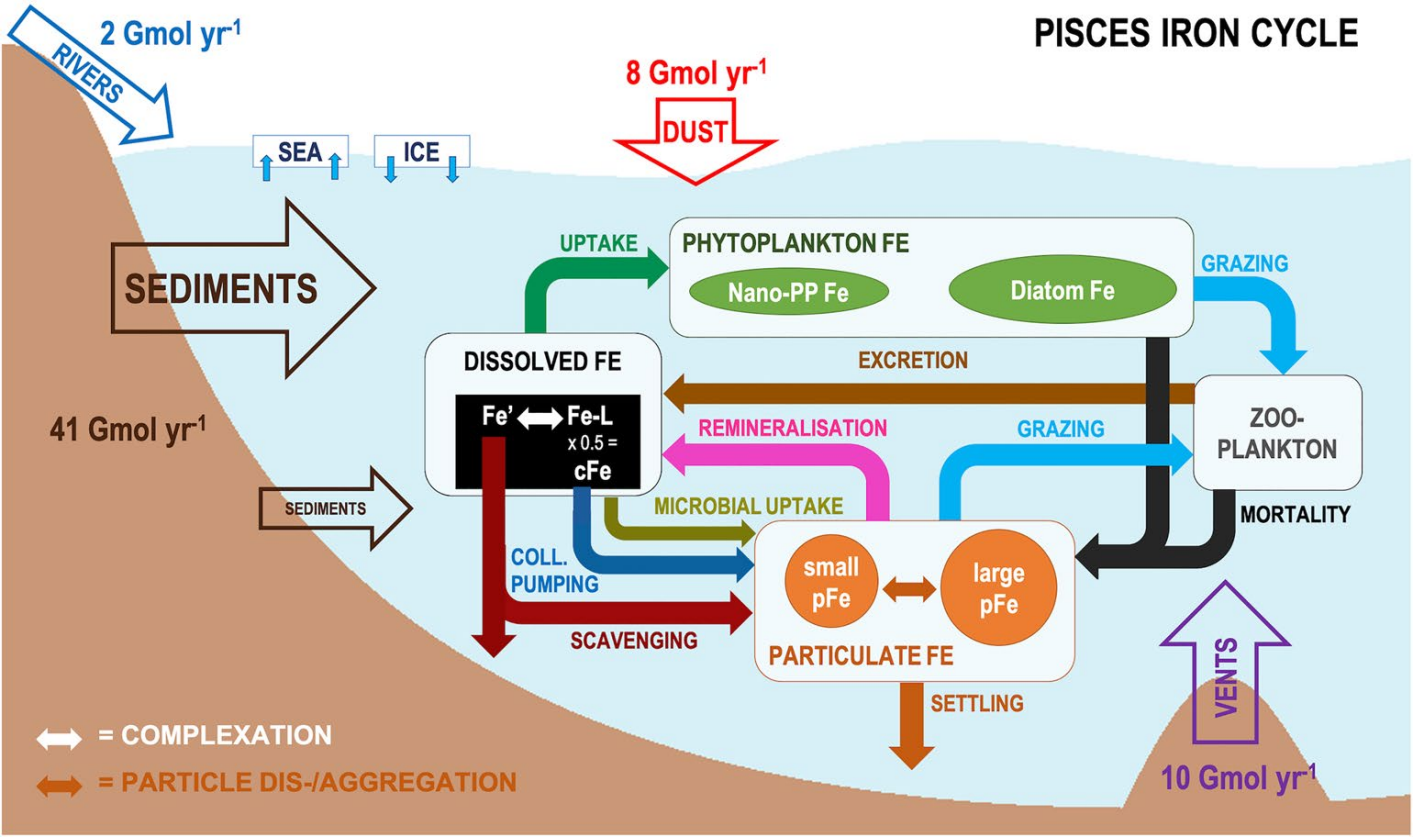
Fe cycle representation in PISCES. The model includes five dynamic Fe tracers (dissolved Fe, Fe in nanophytoplankton and diatoms, small and large Fe particles) and four sources (sediments, dust deposition, hydrothermal systems, and rivers). See Aumont et al. (2015) for a detailed explanation of tracers and processes in this schematic.

The PISCES Fe cycle contains five prognostic Fe tracers (dFe, diatom Fe, nanophytoplankton Fe, small pFe, and large pFe), one dynamic ligand pool, and four external Fe sources (Figure 1). Within the dFe pool, free (Fe′) and organic ligand‐bound Fe (FeL) concentration is determined explicitly assuming equilibrium as a function of the concentration and conditional stability of Fe‐binding ligands (Aumont et al., 2015; Völker & Tagliabue, 2015), whereby 50% of FeL is assumed to be colloidal. Ligands are produced via phytoplankton exudation, zooplankton grazing, and particle degradation. Ligands are removed during phytoplankton uptake of FeL, photochemistry, and bacterial decay accounting for a reactivity continuum (Völker & Tagliabue, 2015). Besides external input, dFe is also produced internally from the remineralization of pFe and recycling by zooplankton. Removal of dFe occurs via abiotical pathways (scavenging and colloidal pumping) and via microbial uptake (by phytoplankton and bacteria). As there are no external sources of particulate Fe (pFe), abiotic transfer from the dissolved to the particulate Fe pool is the main source of pFe, together with Fe losses from zooplankton, and, to a lesser extent, uptake by bacteria (which is added to the pFe pool).

In PISCES, sediments provide the greatest external flux of Fe, followed by hydrothermal vents and dust input. Riverine inputs are locally important, and sea ice is a source or sink in response to sea ice melting and formation, respectively. Sedimentary Fe input ($F_{Fe}^{sed}$, Equation 1) depends on the degree of sediment oxygenation (represented by factor F_sed_) and the fraction of ocean‐bottom area of each cell accounting for high‐resolution topography and enhanced for steep continental slopes (%_sed_), and a maximum input flux ($F_{Fe,max}^{sed}$; Equation 1, from Aumont et al., 2015).

(1) $$F_{\text{Fe}}^{\text{sed}}=F_{\text{Fe,max}}^{\text{sed}}*F_{\text{sed}}*{\%}_{\text{sed}}.$$

**Figure 2:**
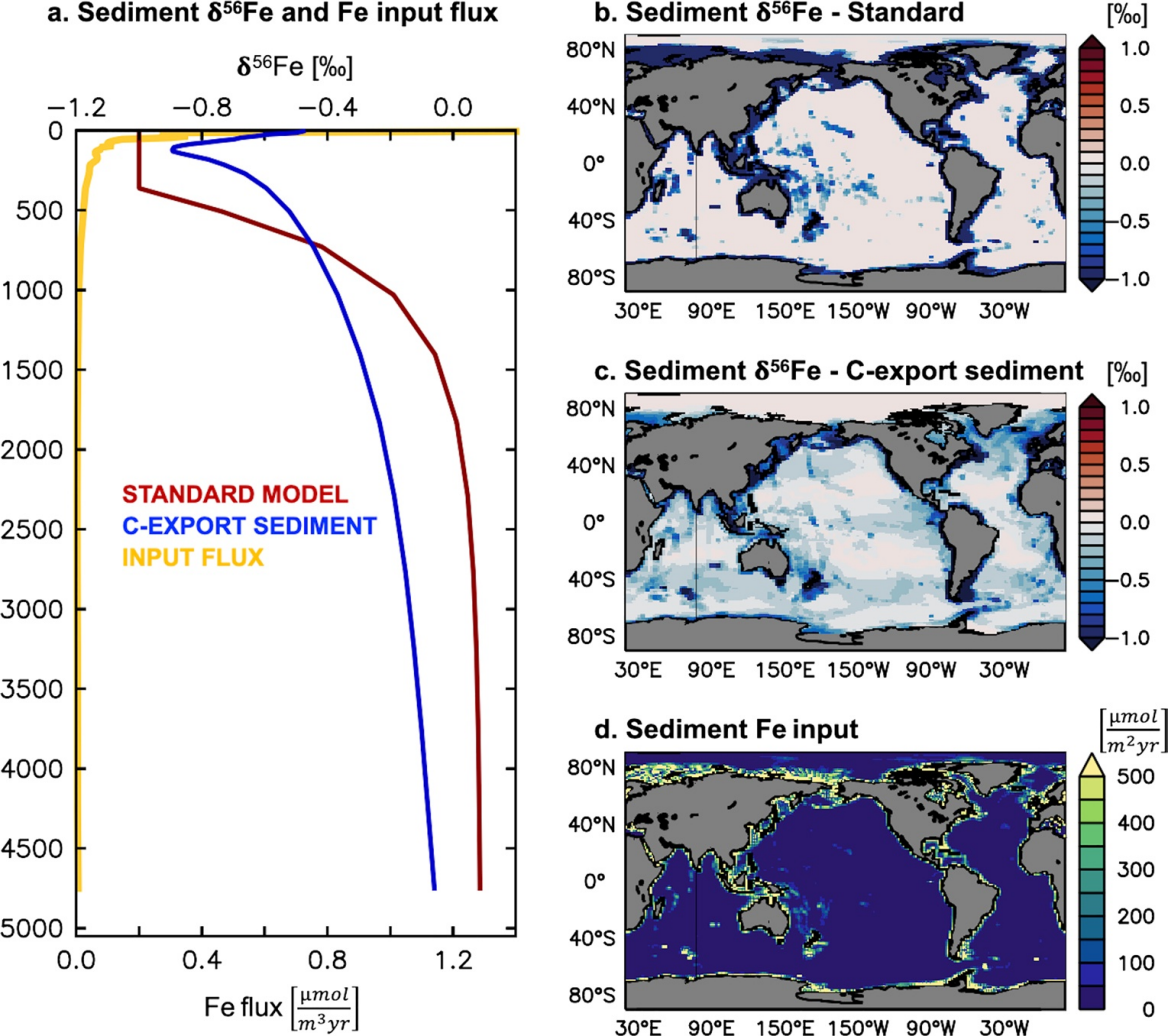
Sediment δ^56^Fe endmember and Fe input flux for the standard experiment and the C‐export sediment experiment. Horizontally averaged sediment δ^56^Fe (‰) and Fe input flux (μmol m^−3^ yr^−1^) with depth (a), depth‐integrated sediment δ^56^Fe (‰) for standard experiment (b) and C‐export sediment experiment (c), and depth‐integrated Fe input (μmol m^−2^ yr^−1^; identical for both experiments; d).

Sediment‐oxygenation factor F_sed_ is simply a function of depth (based on the metamodel of Middelburg et al. [1996]) and its value exponentially decreases from 1 in the upper ca. 400 m to ca. 0.003 at depth. Consequently, sedimentary Fe input is highest for shallow shelves and decreases rapidly with depth (Figures 2a and 2d). Hydrothermal Fe supply is coupled to ridge spreading rates based on OCMIP input fields (Tagliabue et al., 2010), assuming 10^7^ mol Fe/mol He, with concomitant input of 0.5‐fold Fe‐binding ligands (Tagliabue & Resing, 2016). Dust and riverine Fe inputs are identical to Resing et al. (2015) and are described in Aumont et al. (2015).

### 2.2 Introducing Fe Isotopes Into PISCES

We introduced Fe isotopes into PISCES by splitting each of the five Fe tracers into heavy (^56^Fe) and light (^54^Fe) Fe isotope pools (retaining the same total Fe content of each combined Fe tracer), which neglects the much smaller contributions of ^57^Fe and ^58^Fe isotopes. This allows us to calculate the Fe concentration (Equation 2a) and δ^56^Fe (Equation 2b) of each Fe tracer at any given point, in delta notation relative to the commonly used IRMM‐014 isotope standard (ca. 15.67; Meija et al., 2016).

(2a) Fe=Fe56+Fe54,

(2b) δ56Fe[‰]=(Fe56/Fe54FeIRMM−01456/FeIRMM−01454−1)∗1000

In the base Fe isotope model, we multiplied heavy and light isotopes with their respective natural abundances (ca. 94% and 6%; Meija et al., 2016) and ran a 2000‐year spin‐up until both the δ^56^Fe_diss_ and dFe concentration distributions were relatively stable. The dFe concentration field was unchanged from previous experiments with the PISCES model.

We then included the effect of specific source δ^56^Fe endmembers and fractionation during internal cycling. To implement variable source endmembers (Equation (3a), (3b), (3c)), we multiplied the input flux of each Fe source (J_src,Fe_) with the respective fraction of ^56^Fe and ^54^Fe calculated based on a chosen δ^56^Fe endmember (δ^56^Fe_src_; Table 1). This allowed us to determine the flux of heavy (Equation (3a), (3b), (3c)) and light (Equation (3a), (3b), (3c)) Fe isotopes without changing the overall Fe input strength.

(3a) Jsrc,F56e=Jsrc,Fe∗Rsrc,FeRsrc,Fe+1,

(3b) Jsrc,Fe54=Jsrc,Fe∗1Rsrc,Fe+1,

(3c) Rsrc,Fe=FeIRMM−01456FeIRMM−01454∗(δFesrc561000+1).

**Table 1:** Overview of Model Experiments and Their Input Parameters

Experiment	Source δ^56^Fe endmembers (‰)			Fractionation factors	
	Dust	Hydrothermal	Sediments (integrated) *Parameterization of oxygenation*	Complexation	Uptake
Standard	+0.09	−0.5	−1 to +0.09 (−0.80) *Using depth dep F*_*sed*_	1.0006	0.9995
Endmembers only	+0.09	−0.5	−1 to +0.09 (−0.80) *Using depth dep. F*_*sed*_	1	1
Fractionation only	0	0	0	1.0006	0.9995
Light hydrothermal	+0.09	−1.35	−1 to +0.09 (−0.80) *Using depth dep. F*_*sed*_	1.0006	0.9995
Neutral hydrothermal	+0.09	0	−1 to +0.09 (−0.80) *Using depth dep. F*_*sed*_	1.0006	0.9995
Uniform sediment	+0.09	−0.5	−0.80 *Uniform input*	1.0006	0.9995
C‐export sediment	+0.09	−0.5	−2.23 to +0.09 (−0.69) *Using C‐export dep. F*_*sed, Cexp*_ (Equation (3a), (3b), (3c))	1.0006	0.9995

Riverine Fe supply was thereby kept at 0‰, due to insufficient constraints, and sea ice inputs were assumed to have the same δ^56^Fe as the local dFe pool.

To account for isotope fractionation during internal cycling within the ocean, we applied fractionation to Fe complexation by organic ligands and uptake by phytoplankton). We, therefore, split the corresponding process rates (J_proc_) to calculate the transfer of heavy (Equation 4a) and light (Equation 4b) Fe separately, using the fractionation factor of the process (α_proc_; Table 1) and the isotope ratio (R_tra,Fe_) of the Fe tracers that are being converted.

(4a) Jproc,Fe56=Jproc∗Rtra,Fe∗αprocRtra,Fe∗αproc+1,

(4b) Jproc,Fe54=Jproc∗1Rtra,Fe∗αproc+1,

(4c) Rtra,Fe=Fetra56Fetra54.

In the case of Fe complexation, the fractionation factor was applied indirectly to the colloidal pumping process, which removes FeL, and particle scavenging, which removes Fe′. Consequently, the inverse of the fractionation factor (α^−1^) was applied for scavenging of Fe′.

### 2.3 Standard Experiment

For the standard version of the Fe isotope model (“Standard”, Table 1), we used a crustal δ^56^Fe endmember for dust deposition (+0.09‰), a moderately negative value for hydrothermal input (−0.5‰; based on observed vent fluid δ^56^Fe), and negative to crustal values for sedimentary Fe input (−1‰ to +0.09‰; based on the balance of reductive and non‐reductive sedimentary Fe inputs). The δ^56^Fe endmember of sedimentary Fe input is assumed to depend on sediment oxygenation; the lower end of this range reflects input from reductive dissolution processes where oxygenation is low, and the upper‐end crustal input from non‐reductive processes where oxygenation is high (Homoky et al., 2021). We used the depth‐dependent sediment oxygenation factor F_sed_ of the PISCES model (Section 2.1, Aumont et al., 2015) to estimate the respective contributions of reductive and non‐reductive processes to the overall sediment δ^56^Fe endmember (Equation 5). Reductive (δ^56^Fe_RD_) and non‐reductive (δ^56^Fe_NRD_) endmembers in Equation 5 were set to −1‰ and +0.09‰, respectively.

(5) $$\text{Sediment}\;\delta^{56}\text{Fe}=F_{\text{sed}}*\delta^{56}{\text{Fe}}_{\text{RD}}+\left(1-F_{\text{sed}}\right)*\delta^{56}{\text{Fe}}_{\text{NRD}}$$

Using these transitional values, the sediment δ^56^Fe endmember increases from light values for shallow sediments (upper ca. 400 m) to crustal values at depth (below ca. 2,000 m; Figure 2a), without any horizontal variability. As the sedimentary Fe input flux itself also depends on F_sed_ (and decreases with depth), the spatially integrated sediment δ^56^Fe endmember in the model of −0.80‰ is closer to reductive endmember δ^56^Fe_RD_. This overall dominance of reductive input also meant that to avoid unrealistically light δ^56^Fe_diss_ in the water column, our choice of δ^56^Fe_RD_ (−1‰) was at the higher end of porewater observations from reducing sediments (ca. −0.3‰ to −3.4‰; Bergquist & Boyle, 2006; Henkel et al., 2018; Homoky et al., 2009, 2013, 2021; Klar et al., 2017; Severmann et al., 2006, 2010).

Uptake by phytoplankton and complexation by ligands are the only processes in the standard Fe isotope model set to fractionate Fe isotopes. We included preferential uptake of light Fe (α set as 0.9995; same for both size classes) and complexation of heavy Fe (α set at 1.0006). These fractionation factors are taken from observational studies on phytoplankton uptake (Ellwood et al., 2020), and laboratory experiments with relatively strong organic ligands (Dideriksen et al., 2008).

All experiments were run for 350 years to ensure the δ^56^Fe_diss_ and dFe concentration distributions were stable.

### 2.4 Model Sensitivity Experiments

We ran a suite of sensitivity experiments branched from the standard experiment to examine different aspects of the Fe isotope cycle (Table 1).

To study the relative importance of external source endmembers and the fractionation by internal cycling, we set up two experiments where either fractionation was turned off (i.e., both fractionation factors set to 1; “Endmembers only”), or where all endmembers were set to 0‰ (“Fractionation only”).

To assess how endmember assumptions affected the large‐scale impact of hydrothermal vents on the global distribution of δ^56^Fe_diss_, we tested two different hydrothermal δ^56^Fe endmembers. The “Light hydrothermal” experiment was significantly lighter than our standard value at −1.35‰ and the “Neutral hydrothermal” experiment used an endmember of 0‰.

To examine how different controls on the sediment δ^56^Fe endmember affected the global distribution of δ^56^Fe_diss_, we set up two experiments with either uniform sediment endmember of −0.8‰ at all depths (“Uniform sediment”; set to match the integrated sediment endmember value of the standard experiment), and we developed a new organic carbon‐export dependent sediment endmember parametrization (“C‐export sediment”). We used the latter to test if export of carbon to sediments is a better proxy for sediment oxygenation, and consequently the reductive release of light Fe, than the depth‐dependent oxygenation factor F_sed_ of the standard experiment. We, therefore, replaced F_sed_ (in Equation 5) with the carbon‐export dependent term F_sed, Cexp_ (Equation 6), so that the reductive contribution to the sediment endmember was highest for sediments receiving high seafloor organic carbon‐export fluxes (C_exp_) as per Equation 6.

(6) $$F_{\text{sed,Cexp}}=\frac{\text{Cexp}}{K_{\text{Cexp}}+C_{\text{exp}}},$$

The half‐saturation constant K_Cexp_ was set to 60 nmol m^−2^ s^−1^. As for the depth‐dependent F_sed_ (Section 2.1), the newly introduced F_sed,Cexp_ varies between 0 and 1, but unlike F_sed_, it rarely reaches 1 (i.e., 100% reductive input)—except in a few locations with very high export fluxes. Consequently, this parameterization required a lower, and likely more realistic, reductive endmember (δ^56^Fe_RD_; Equation 5), which was set to match the δ^56^Fe determined for reductive Fe fluxes in a California margin basin (−2.4‰; John et al., 2012). Spatially integrated, the sediment δ^56^Fe endmember of the C‐export sediment experiment was slightly less negative (−0.69‰) than for the standard experiment (−0.80‰), whereas its range of sediment endmembers was larger (Table 1). It also permitted horizontal variations, with the lightest sediment endmember values found underlying productive margins (Figure 2). Importantly, we only adapted the δ^56^Fe endmember parameterization; the sedimentary Fe input flux itself remained depth‐dependent for all experiments.

### 2.5 Observational Constraints

Model performance was assessed against δ^56^Fe_diss_ and dFe concentration observations of a global data set, as well as by focusing specifically on five full‐depth GEOTRACES sections (referred to as “GEOTRACES section data set”). The GEOTRACES section data set consists of three Atlantic sections: GA03w (referred to as “GA03”; Conway & John, 2014; Hatta et al., 2015), GA10 (Conway et al., 2016; Schlitzer et al., 2018; Summers, 2020), and GIPY04 (Abadie et al., 2017; Chever et al., 2010), and two in the Pacific: GP16 (Fitzsimmons et al., 2017; John et al., 2018; Resing et al., 2015), and GP19. The “global” data set further includes additional data from SW Pacific (Ellwood et al., 2015, 2020; Labatut et al., 2014; Radic et al., 2011), SE Pacific (Chever et al., 2015; Fitzsimmons et al., 2016), North Pacific (Conway & John, 2015; John et al., 2012; Pinedo‐González et al., 2020), North Atlantic (John & Adkins, 2012; Klar et al., 2018), Southern (Ellwood et al., 2020; Lacan et al., 2008; Sieber et al., 2021), and Arctic Ocean (Charette et al., 2020). Data from studies specifically targeting small‐scale features such as (near‐field) hydrothermal plumes were not included. We extracted the Fe isotope and concentration data for GA03, GIPY04, GP16, most of GA10, and some of the additional global observations from the GEOTRACES IDP2017 (Schlitzer et al., 2018), and augmented it with new data from GP19 (to be published in the GEOTRACES IDP2021). Typical analytical uncertainties associated with dissolved Fe isotope measurements below the surface are 0.04‰–0.07‰ (e.g., Conway et al., 2016), with larger uncertainties typical of surface waters.

For the visual assessment of the GEOTRACES section data set, model output was extracted at each observational station of each section and at intermediate grid points where stations were far apart. Observations were binned onto model depth levels (see Section 2.1), except for the uppermost 16 depth layers (upper 197 m), where two layers were combined at a time for increased visibility in the figures. For the quantitative assessments, we binned the observational data sets into a 1° × 1° common horizontal grid and onto the model vertical grid (see Section 2.1) and conducted linear regressions between observation and model data using identical spatial coordinates and model outputs from the month the observations were collected. For dFe concentration, we only used data points for which δ^56^Fe_diss_ data was available and performed a log transformation before the regression. We quantified the agreement between the model and observations using the correlation coefficient (*R*), root mean squared error (RMSE), slope and intercept of the regression, as well as the mean and standard deviation (SD) of observations and extracted model values.

## 3 Results and Discussion

### 3.1 Assessment of the Standard Experiment

**Figure 3:**
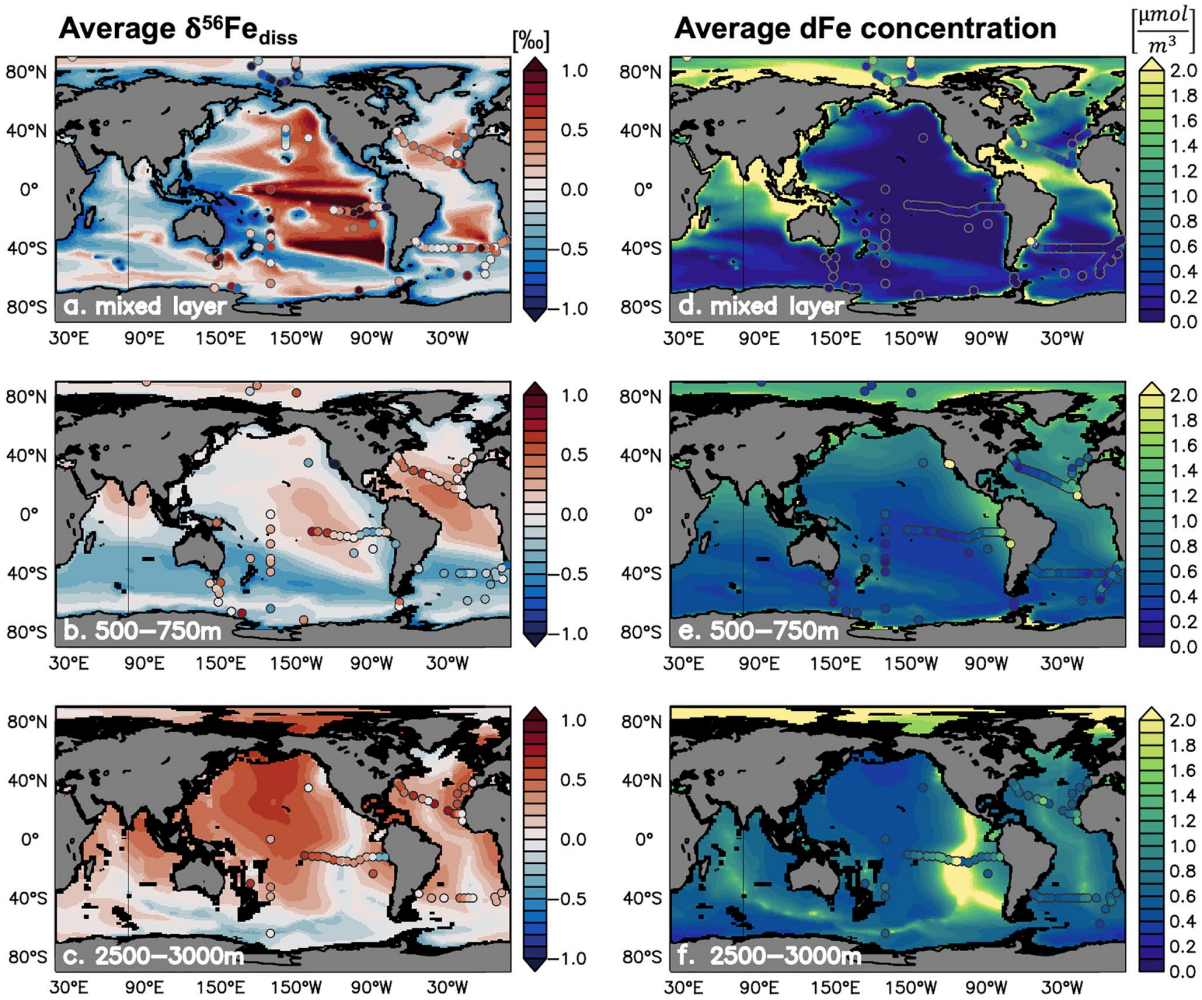
Standard experiment δ^56^Fe_diss_ (a–c; ‰) and dFe concentration (d–f; μmol m^−3^) averaged over the mixed layer (a, d; local annually averaged mixed layer depth), 500–750 m (b, e), and 2,500–3,000 m depth (c, f). Available observations within each depth layer are plotted on top as filled circles without averaging.

The standard experiment (Table 1) has a broadly similar skill in reproducing δ^56^Fe_diss_ as for dFe concentration against the global data set (Table 2). In the surface ocean, modeled δ^56^Fe_diss_ varies substantially, with light signatures (<−0.5‰) in waters influenced by continental margins to increasingly heavy values (>+1‰) in parts of the open ocean (Figure 3a). Observations show similarly systematic variations in δ^56^Fe_diss_ and match the model well in certain areas (e.g., Eastern equatorial Pacific), but are less well aligned in others (e.g., SW Pacific). At intermediate depths (500–750 m), δ^56^Fe_diss_ values are less variable (Figure 3b) and generally lighter than in deeper layers (2,500–3,000 m, Figure 3c) by around −0.1‰. There also appears to be a North‐South gradient in the 500–750 m depth stratum, with values increasing from south to north, especially in the Atlantic (Figure 3b). These broad patterns also agree well with observations, but there are discrepancies close to some margins, where the modeled values are either lighter (e.g., SW Pacific), or heavier (e.g., Eastern equatorial Pacific, NE Atlantic) than observed. There is little data available at present for the North Pacific, but the SAFe station at 30°N 140°W displays a light δ^56^Fe_diss_ signal in the intermediate water depth range (Conway & John, 2015), that is not reproduced by the model (Figure 3b). In the deep ocean (2,500–3000 m), modeled δ^56^Fe_diss_ is generally heavy, with negative values restricted to recently ventilated waters at high latitudes and close to hydrothermal vent inputs (Figure 3c). Again, while the agreement with observations is generally good, it is notable that the model does not reproduce the light values observed close to the eastern boundary continental margins where observations are available (Figure 3c). In contrast, the model appears to overestimate the impact of hydrothermal vents on δ^56^Fe_diss_, especially around the South East Pacific Rise (SEPR).

**Table 2:** Statistical Performance of Model Experiments Against the Global Data Set

Parameter	Experiment	Nr.	Mean	SD	RMSE	R	Slope	Intercept
Log dFe concentration	Observations^a^	1,173	−0.50	0.49				
	Model^b^		−0.26	0.52	0.48	0.65	0.69	0.09
δ^56^Fe_diss_	Observations	1,197	+0.12‰	0.52‰				
	Standard		+0.01‰	0.36‰	0.51‰	0.40	0.27	−0.03
	Endmembers only		−0.59 ‰	0.26‰	0.85‰	0.48	0.24	−0.62
	Fractionation only		+0.60‰	0.23‰	0.73‰	0.09	0.04	0.59
	Light hydrothermal		−0.10 ‰	0.36‰	0.57‰	0.32	0.22	−0.13
	Neutral hydrothermal		+0.07‰	0.39‰	0.51‰	0.40	0.31	0.03
	Uniform sediment		+0.06‰	0.31‰	0.51‰	0.33	0.19	0.03
	C‐export sediment		+0.15‰	0.41‰	0.65‰	0.03	0.03	0.15

^a^ For same locations as δ^56^Fe_diss_ data (if available).

^b^ Same dFe model for all experiments.

**Figure 4:**
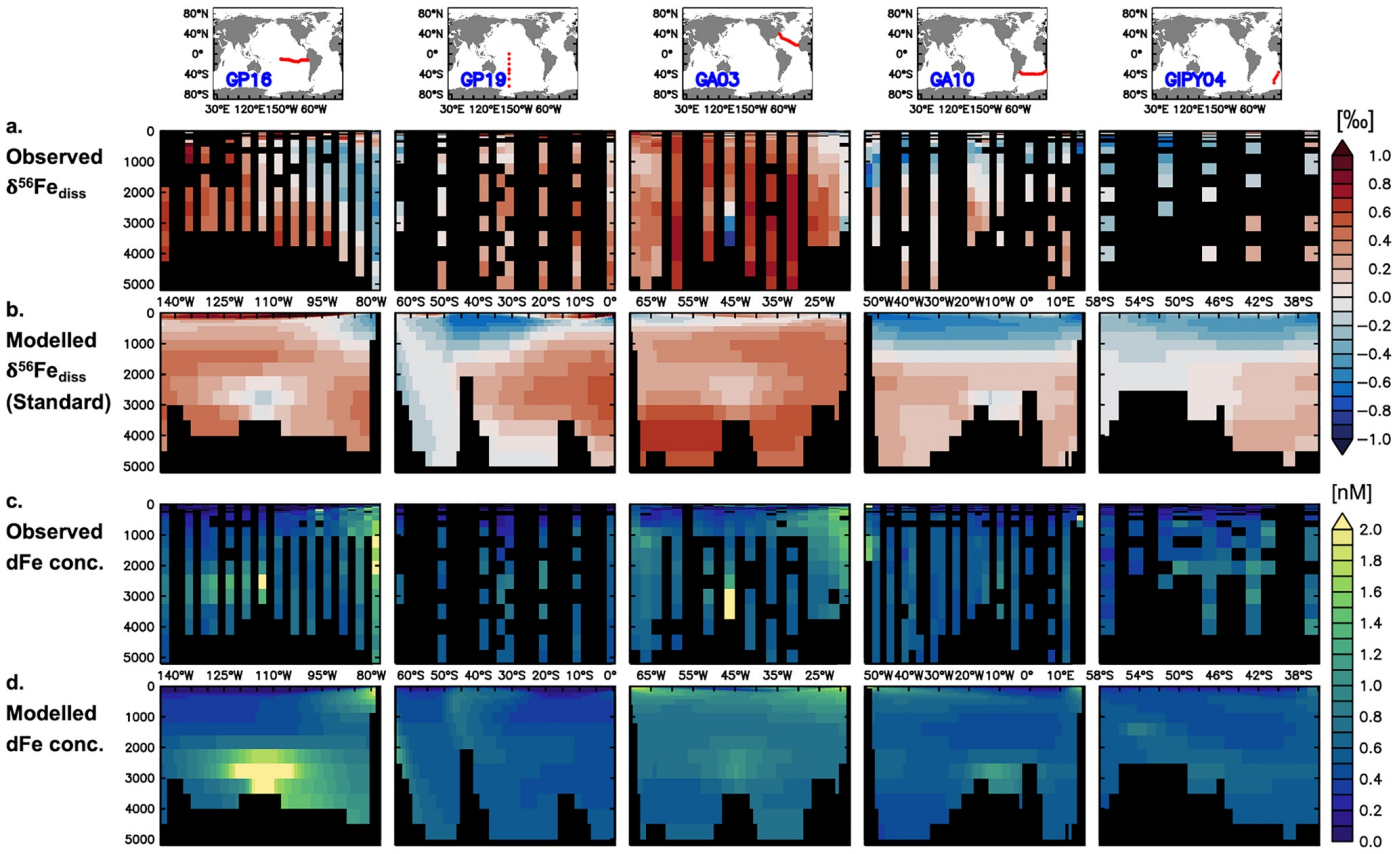
Observed (a, c) and standard experiment (b, d) δ^56^Fe_diss_ (a, b; ‰) and dFe concentration (c, d; μmol m^−3^) for five GEOTRACES sections. Data are binned at model depths; model output is extracted at the same coordinates as observations, and on interpolated coordinates in between.

Focusing in detail on five GEOTRACES sections also highlights the performance of the model and the potential role of different mechanisms governing the oceanic distribution of δ^56^Fe_diss_ (Figures 4a and 4b). Large‐scale trends in the observations, such as lighter signatures in the southern parts of basins are reproduced, and the model captures the vertical δ^56^Fe_diss_ gradient observed on the South Atlantic GA10 section very well. Similar discrepancies as for the global data set are evident close to certain continental margins, where modeled light δ^56^Fe_diss_ anomalies are either too extensive (GP19, ca. 40°S; GA03, western margin), or too limited (GP16, GA03, and eastern margins). The modeled vertical extent of light anomalies also appears too restricted on eastern margins, whereas for hydrothermal vents, light anomalies are often larger than observed (GA03, TAG field at ca. 45°W; GP16, SEPR at ca. 117°W; GA10, 30°–0°W). Some of these discrepancies are also evident in the dFe concentration distribution (Figures 4c and 4d), as the model appears to overestimate release from some vents, but underestimates input from certain deep sediments. In other cases, the coarse resolution of the model may preclude the reproduction of synoptic features often targeted on GEOTRACES sections, such as near‐field hydrothermal plumes or processes operating on seasonal or sub‐seasonal timescales in the upper ocean.

### 3.2 Quantifying the Relative Role of Source δ^56^Fe Endmembers and Internal Fractionation Processes

**Figure 5:**
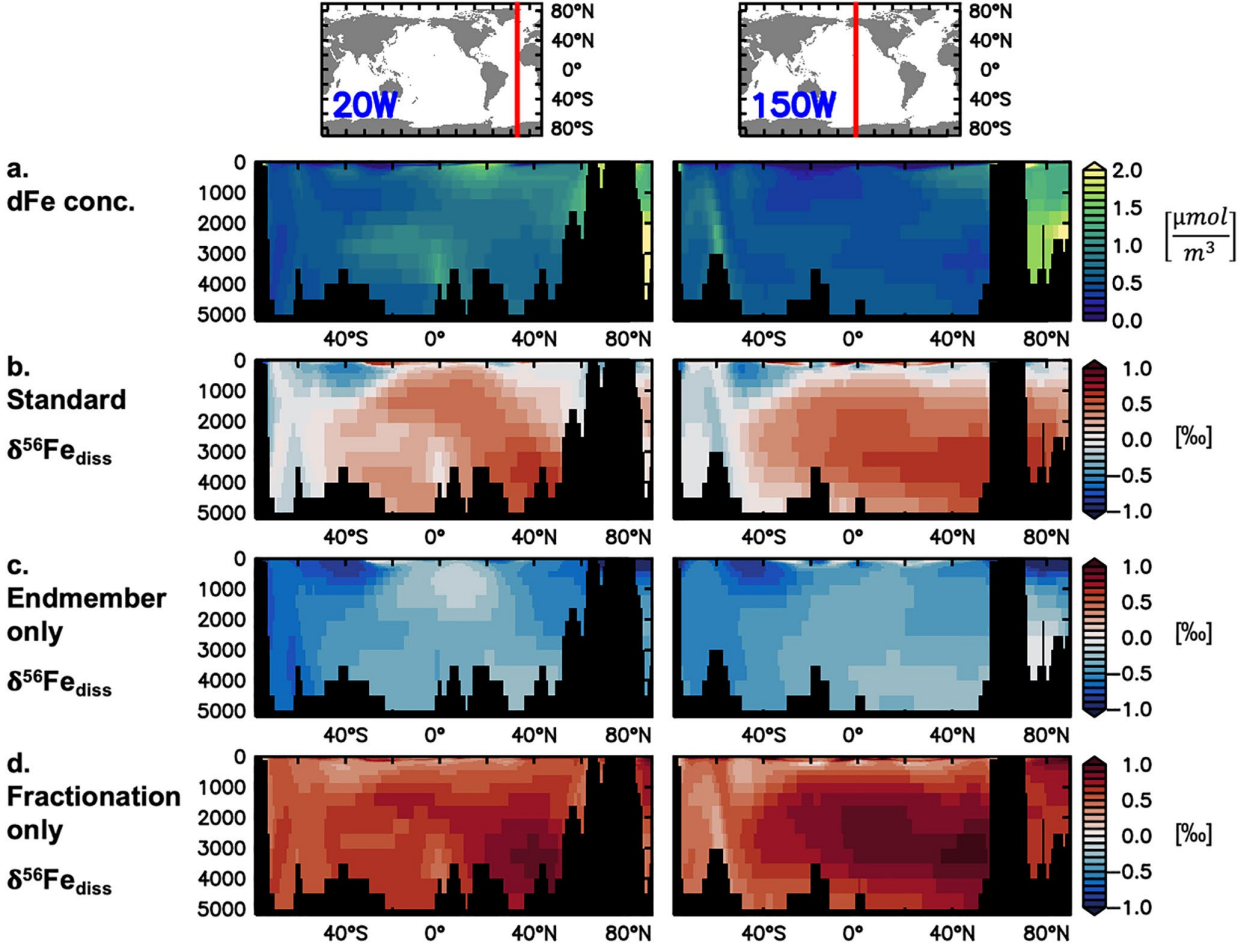
Modeled dFe concentration (a; μmol m^−3^; same for all experiments) and δ^56^Fe_diss_ (‰) of the standard (b), endmembers only (i.e., no fractionation; c), and fractionation only (i.e., no source‐specific endmembers; d) experiments for two meridional sections in the Atlantic and Pacific Oceans (20°W and 150°W, respectively).

Experiments with altered isotopic fractionation factors and δ^56^Fe endmembers allowed us to dissect their relative roles in shaping the δ^56^Fe_diss_ distribution in the standard version of the Fe isotope model. Given the generally good performance of the standard experiment, we here assess the role of distinct processes using the modeled δ^56^Fe_diss_ along two meridional sections at 20°W and 150°W to represent the broad distributions in the Atlantic and Pacific Oceans, respectively (Figure 5). Overall, a combination of variable source δ^56^Fe endmembers and isotopic fractionation governs the modeled trends in δ^56^Fe_diss_ (Figures 3, 4, and 5b) and sensitivity experiments without fractionating processes and those with neutral endmembers produce δ^56^Fe_diss_ distributions (Figures 5c and 5d) that are clearly mismatched with those observed (Table 2; e.g., means, intercept).

Turning off isotopic fractionation during internal cycling and only considering δ^56^Fe endmember signals leads to negative δ^56^Fe_diss_ for almost the entire water column (Figure 5c), in contrast to the majority of observations. Values are lightest in upper ocean areas where reductive sedimentary Fe input dominates (e.g., at around 40°S), as well as in well‐ventilated deep ocean areas and sites close to hydrothermal vents. Input from dust leads to close to crustal δ^56^Fe_diss_ in parts of the upper ocean, especially where dust supply is dominant over other sources, and at intermediate depths in response to subsurface dissolution and remineralization of dust‐derived Fe (e.g., around 20°N). A combination of crustal input from oxic sediments and lack of ventilation leads to similarly near‐crustal values for older waters in the ocean interior. The heavy δ^56^Fe_diss_ values measured in parts of the surface ocean (up to +2‰; Figure 3a, obs.) and at depth (>+0.2‰ below 2,000 m, on average; Figure 3c, obs., Figure 4a) cannot be reproduced unless these source endmember values were altered well outside the accepted range.

Isotopic fractionation during both phytoplankton uptake and organic complexation of dFe is key for the emergence of heavy δ^56^Fe_diss_ values in our model (Figure 5d). Heavy dFe is preferentially complexed by organic ligands, leaving behind lighter, uncomplexed Fe′ that is continuously removed by scavenging. Consequently, δ^56^Fe_diss_ values progressively increase in the ocean interior, especially in the absence of any external inputs of light dFe. Preferential uptake of light dFe by phytoplankton mainly affects the surface ocean (upper ca. 200 m), where it can lead to increasingly heavy δ^56^Fe_diss_ (Figure S2), especially in areas where Fe is limiting (e.g., Equatorial Pacific or Southern Ocean). In certain areas, this can also affect deeper water layers, due to subduction of waters, from the Southern Ocean in particular, enriched in heavy Fe, as well as by the remineralization of Fe from biomass (Section 3.3). At high latitudes, the strong seasonality in phytoplankton Fe uptake leads to a seasonal pattern in surface δ^56^Fe_diss_, with heavier values in summer months than in winter (Figure S3). Overall, our model results clearly point to the role of fractionation in reproducing the heavy δ^56^Fe_diss_ signals observed in the surface and deep ocean (Figures 3 and 4).

Ultimately, the δ^56^Fe_diss_ distribution in the standard experiment arises from a combination of external inputs and fractionation processes. For instance, the meridional and vertical gradients in the Atlantic result from distinct source δ^56^Fe endmembers, isotopic fractionation during organic complexation, and water mass subduction. Similarly, heavy surface δ^56^Fe_diss_ in certain open ocean areas are due to uptake fractionation in the absence of light external dFe sources.

### 3.3 Impact of Remineralization and Abiotic dFe Removal Processes Through the Water Column

**Figure 6:**
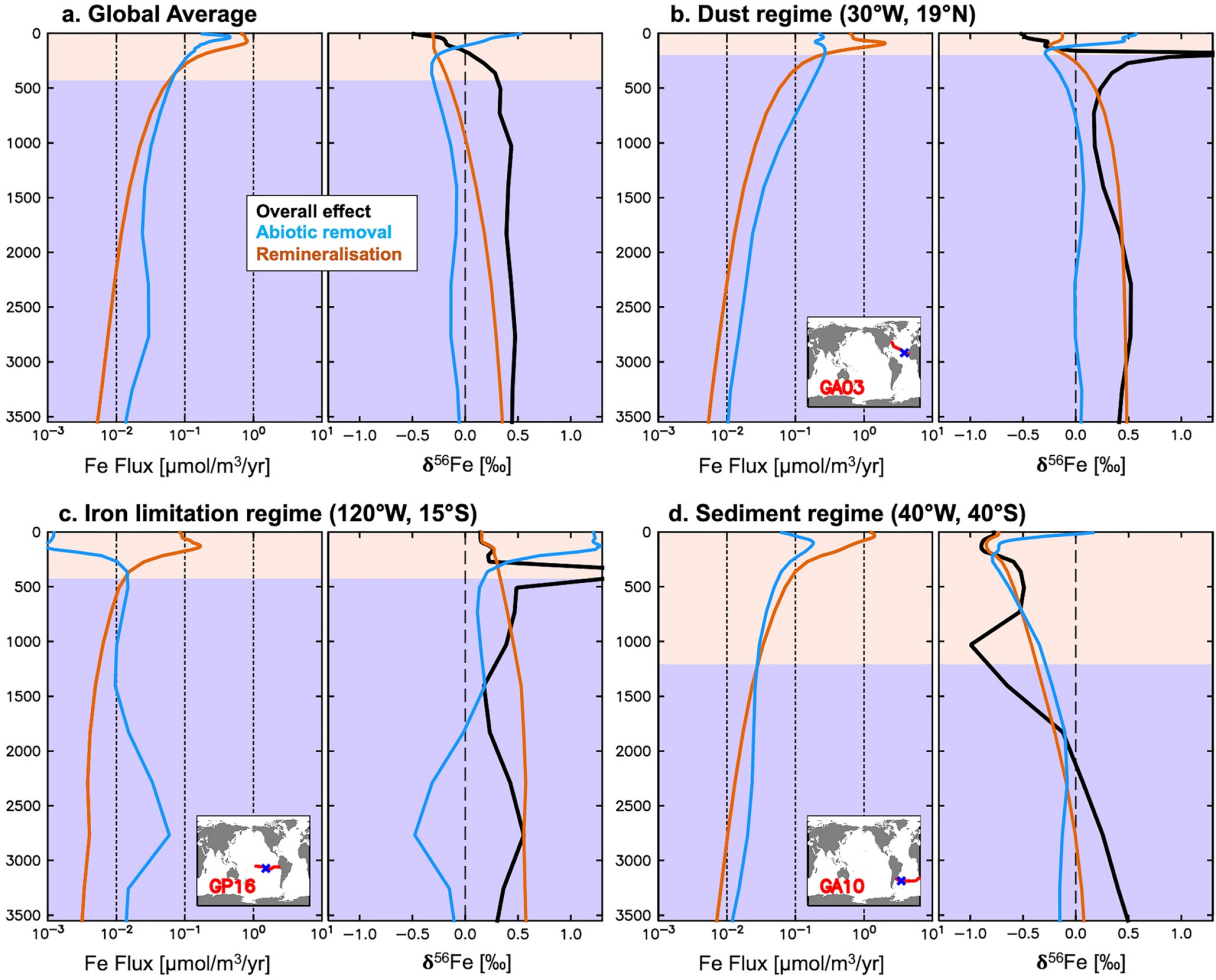
Remineralization (orange) and abiotic removal (blue) Fe fluxes (μmol m^−3^ yr^−1^) and their δ^56^Fe composition (‰) in the standard experiment; global average vertical profile (a) and profiles from three selected locations (b–d). Net effect on δ^56^Fe_diss_ (‰) shown in black. Points with net fluxes below a threshold of ±5 nmol m^−3^ yr^−1^ were excluded before calculating the global average net effect, as δ^56^Fe values calculated from such very small Fe pools are at times extremely high and thus distort the average. Background colors indicate the dominance of remineralization (orange) and abiotic removal fluxes (blue), respectively.

To examine how internal cycling in the ocean interior affects δ^56^Fe_diss_, we focused on the dominant processes remineralization and abiotic removal (the sum of scavenging and colloidal pumping) in our standard experiment. The two distinct abiotic removal processes have opposite effects on δ^56^Fe_diss_ due to the complexation fractionation we included, as scavenging removes light dFe (in the form of Fe′), while the aggregation of FeL by colloidal pumping acts on heavy dFe (Section 2). The impact of remineralization of particles is more complex, as the isotopic composition of the particles can vary in space and time. However, in general, pFe will be isotopically lighter than dFe, due to the enforced fractionation in favor of light Fe by biological cycling and the aforementioned scavenging of light dFe in our model (Section 2). Thus, based on the setting of the two fractionation factors, we expect remineralization and abiotic removal to exert opposing effects on δ^56^Fe_diss_, so that the net effect crucially depends on the (relative) magnitude of the two fluxes. Another key factor is the upper ocean δ^56^Fe_diss_ as it strongly impacts the δ^56^Fe signature of pFe to be remineralized. We, therefore, assessed the balance between remineralization and abiotic removal for three distinct upper ocean δ^56^Fe_diss_ regimes, all located on GEOTRACES transects, as well as on a global scale (Figure 6). Two of the three “case studies” are from locations with one dominant external Fe source (dust and sedimentary Fe, respectively), whereas the third represents a case of severe Fe limitation. For this purpose, we extracted the magnitude and δ^56^Fe of the two fluxes from the standard experiment as a function of depth (noting that where fluxes are small, δ^56^Fe values can be very large).

To diagnose the net effect of remineralization and abiotic removal on δ^56^Fe_diss_, we distinguished between points of net gain (i.e., remineralization > abiotic removal) and of net loss (i.e., remineralization < abiotic removal). For points with “net gain,” this diagnosed effect was based on the δ^56^Fe of added Fe (Equation (3a), (3b), (3c)), calculated from the heavy and light remineralized (^56^Fe_remin_, ^54^Fe_remin_) and removed (^56^Fe_removal_, ^54^Fe_removal_) Fe pools. For points with “net loss,” the diagnosed effect on δ^56^Fe_diss_ was the equal opposite value of “net gain,” as that net Fe pool is being removed (Equation (3a), (3b), (3c)).

(7a) δ56Fenet,gain=(Feremain56−Feremoval56Feremain54−Feremoval54/FeIRMM−01456FeIRMM−01454−1)∗1000,

(7b) δFenet,loss56=−δFenet,gain56.

Looking at the global average vertical profile from the standard experiment, remineralization dFe fluxes exceed abiotic removal in the upper ca. 500 m of the water column, with net abiotic removal of dFe predominant below (Figure 6a). The two processes have the opposite effect on δ^56^Fe_diss_ as remineralization tends to release light dFe (where the flux is substantial), even without any specific fractionation associated with the process of remineralization (Figure S4), whereas abiotic removal removes light dFe (below the uppermost 110 m) because scavenging dominates over colloidal pumping (Figure S5). The net effect on δ^56^Fe_diss_ is thus negative in the upper ca. 170 m, but rapidly increases to about +0.34‰ at 500 m depth when abiotic removal becomes more important (Figure 6a). However, the effect of internal cycling is far from uniform across the ocean in our model and can diverge substantially from this average view.

Variability in the role of remineralization and abiotic removal emerges regionally in our model across different Fe cycling regimes. In the North Atlantic, high dust input of dFe with a crustal δ^56^Fe endmember leads to increased abiotic removal of dFe with mostly crustal signatures, and also fuels remineralization of relatively heavy pFe (Figure 6b). Again, these two processes compensate for each other in the model, leading to a net effect that broadly resembles the global average. The transition between the dominance of remineralization and abiotic removal occurs at a shallower depth (ca. 200 m), and coincides with a very heavy net δ^56^Fe signal that has little impact since absolute fluxes are low here (Figure 6b). Given the heavy net δ^56^Fe below this transition, internal cycling likely contributes to the generally heavy δ^56^Fe_diss_ observed (Figure 4a, GA03). In the South Pacific Fe‐limited regime, the combination of Fe‐limited surface waters and lack of an isotopically light external dFe source leads to heavy dFe values, which then drives the uptake and remineralization of relatively heavy Fe (ca. +0.5‰) throughout the water column (Figure 6c). Under such Fe‐limited conditions, Fe removed by abiotic processes results in heavy δ^56^Fe_diss_ values in surface waters, where colloidal pumping dominates in the model (Figure S5). The influence of colloidal pumping becomes smaller at intermediate depths, and the greater importance of scavenging leads to a profile similar to the global average. In deeper waters (ca. 2,500–3,000 m), there is a transition to a net isotopically light sink as there are large scavenging fluxes removing light Fe from the SEPR hydrothermal plume at this location (Figure 6c). As for the dust regime, the net effect on δ^56^Fe_diss_ for the Fe‐limited regime is isotopically heaviest at the net dFe release/removal transition at ca. 500 m, and remains heavy beneath this transition. This may contribute to the heavy δ^56^Fe_diss_ observed west of the SEPR (Figure 4a, GP16). We observe a markedly different impact in areas such as the SW Atlantic, where light sedimentary Fe input dominates in the upper ocean (Figure 6d). Here, the δ^56^Fe of remineralized Fe is negative at nearly all depths and particularly light (<−0.5‰) in the upper ca. 1000 m where remineralization is dominant. Despite scavenging removal of isotopically light dFe, the net effect on δ^56^Fe_diss_ only becomes positive below ca. 2,000 m depth, once local remineralization fluxes diminish (Figure 6d). Thus, the light net δ^56^Fe in the upper 2,000 m likely contributes to the generally light δ^56^Fe_diss_ observed in this depth layer (Figure 4a, GA10). Overall, globally, there is a variable effect of remineralization and abiotic removal on the cycling of Fe isotopes in our standard model. The influence of these processes on δ^56^Fe_diss_ depends not only on the δ^56^Fe of the Fe that is transferred between Fe pools, but also on the gross removal and release fluxes, which can vary horizontally and vertically. Equally, it must be borne in mind that lateral transport of isotopic signatures can also affect the overall vertical profile of δ^56^Fe_diss_ observed.

### 3.4 Large‐Scale Hydrothermal Impact

**Figure 7:**
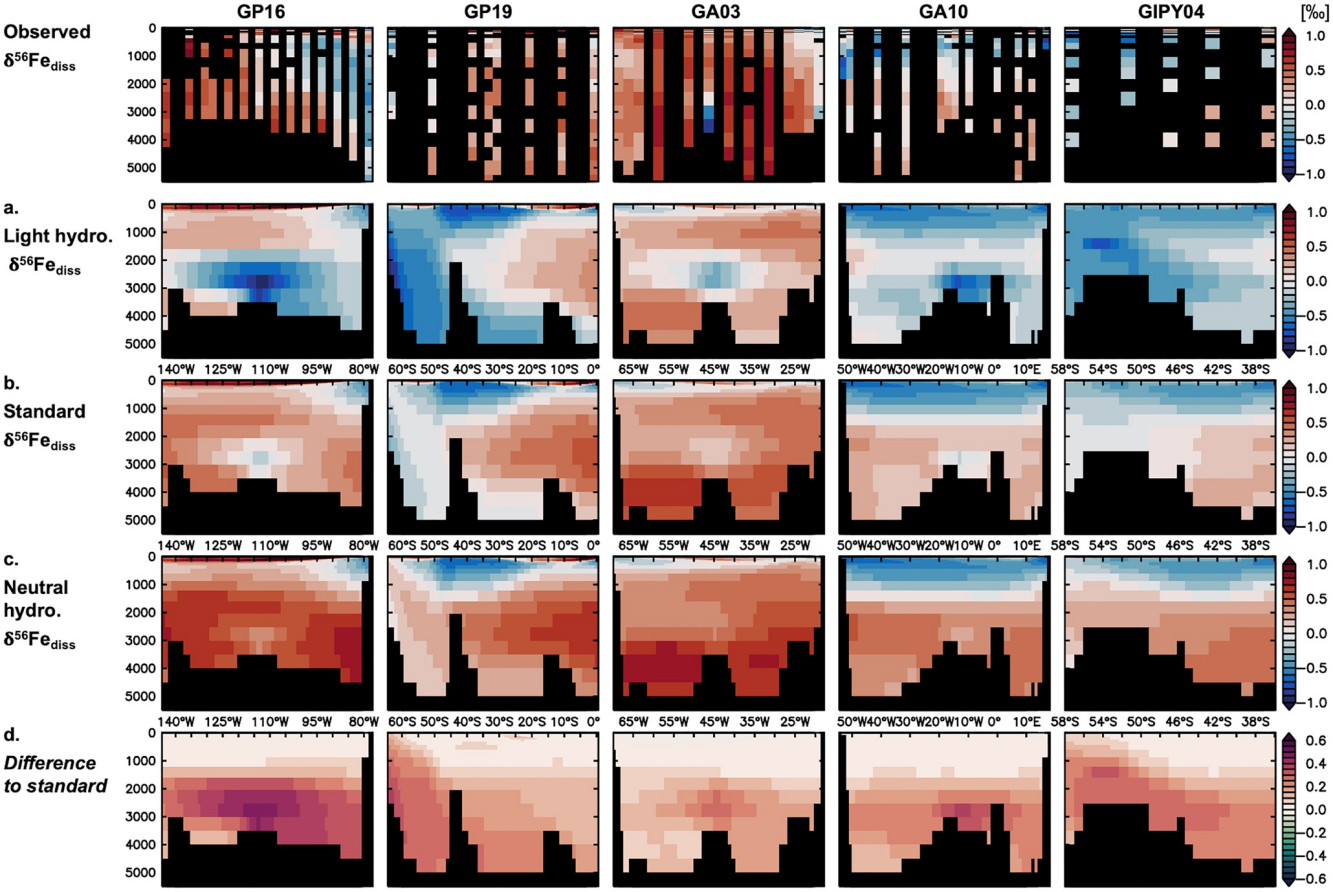
Observed and modeled (a–c) δ^56^Fe_diss_ (‰) for experiments with different hydrothermal δ^56^Fe endmembers for five GEOTRACES sections: (a) light hydrothermal (hydro. δ^56^Fe = −1.35‰), (b) standard (hydro. δ^56^Fe = −0.5‰), and (c) neutral hydrothermal (hydro. δ^56^Fe = 0‰), and (d) the difference between the neutral hydrothermal and standard experiments.

The very light δ^56^Fe_diss_ measured in close proximity to certain vents (e.g., −1.35‰ for TAG) are likely not representative of large‐scale hydrothermal input (in the Atlantic at least) as applying a similarly light hydrothermal δ^56^Fe endmember results in large‐scale disagreement in the ocean interior (Figure 7a). This indicates that stabilization by ligands drives a heavier δ^56^Fe_diss_ signal away from the vent site or the removal of light dFe in the proximal plume must be stronger than currently accounted for in our model. The best overall agreement for the global data set is achieved with either a moderately negative (−0.5‰, standard) or neutral (0‰) hydrothermal endmember (Table 2). Focusing on the GEOTRACES sections, the moderately negative endmember used in the standard experiment appears to be more appropriate for stations influenced by (some) Southern Ocean vents (GIPY04, GP19 south of ca. 40°S; Figures 7b and 7d), whereas a neutral value performs better further north, for example, for the North Atlantic GA03 section (Figures 7c and 7d). This suggests that the large‐scale hydrothermal impact on δ^56^Fe_diss_ differs between vent systems beyond the overall influence of organic complexation alone and that there is likely no single endmember value. Such inter‐site variability has been observed before (mostly for near‐field plumes) and may be caused by variations in vent chemistry and/or interaction with organic material (e.g., Bennett et al., 2009; Fitzsimmons et al., 2017; Lough et al., 2017, 2019), which would affect the hydrothermal Fe input strength, δ^56^Fe composition, and dFe stability across different spatial scales.

### 3.5 Controls on Sediment Endmember

**Figure 8:**
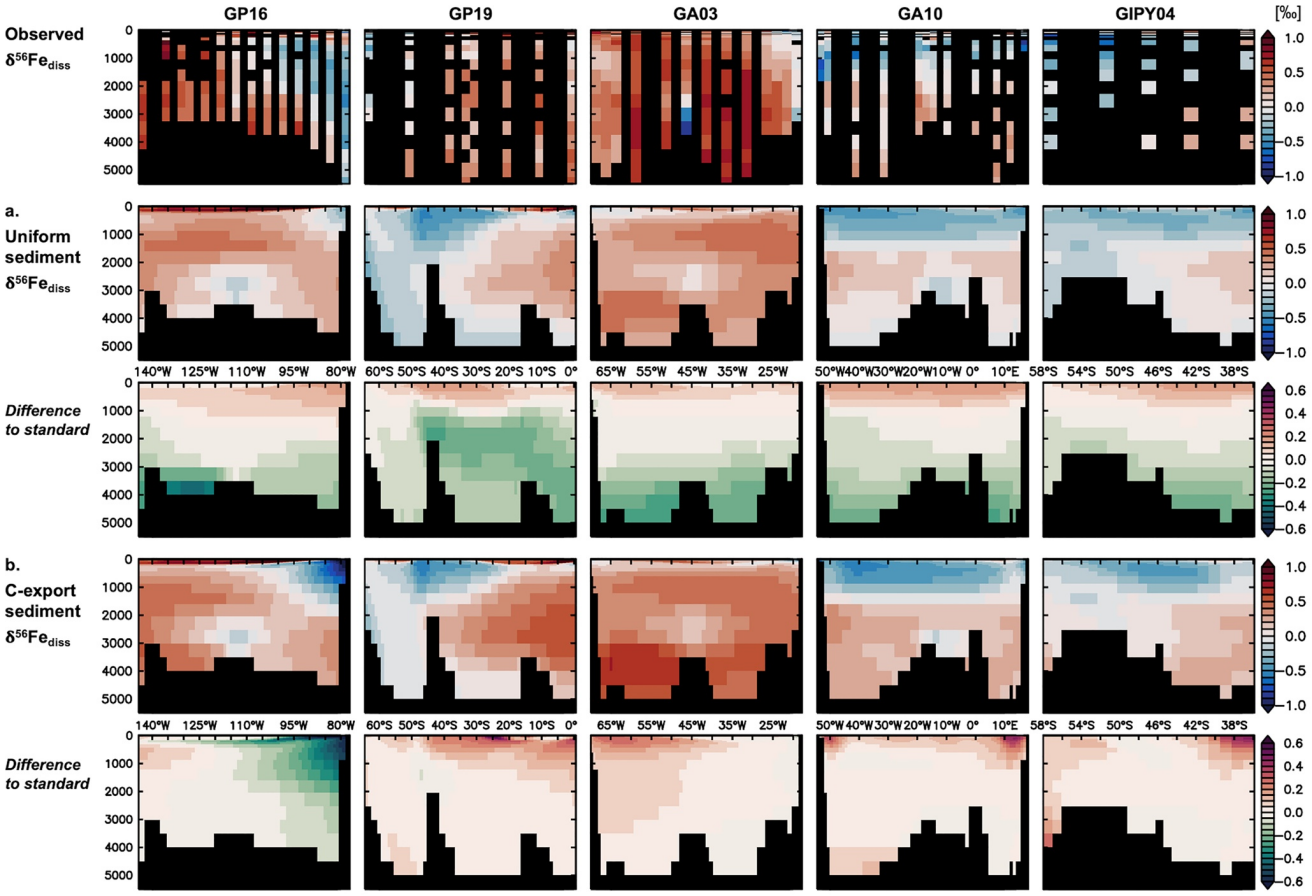
Observed and modeled (a, b) δ^56^Fe_diss_ (‰) for experiments with different sediment δ^56^Fe endmembers for five GEOTRACES sections and their difference to the standard experiment: (a) uniform sediment (sed. δ^56^Fe = −0.8‰), (b) C‐export sediment (sed. δ^56^Fe = −2.23‰ to +0.09‰).

The standard experiment with its oxygenation‐dependent (F_sed_) sediment δ^56^Fe endmember (Equation (3a), (3b), (3c)) performs well in many locations (Figures 3 and 4), and generally agrees better with observations than an experiment with uniformly negative sediment endmember of −0.8‰ (Figure 8a, Table 2). This is especially clear in the ocean interior (Table S1), where a uniformly negative endmember leads to δ^56^Fe_diss_ values that are lighter than for the standard experiment (for which the sediment endmember is crustal below ca. 2,000 m, Figure 2a) and most deep ocean observations (Figure 8a). This underscores previous assertions that a focus on only reductive Fe dissolution processes neglects the input of Fe from deep ocean sediments via non‐reductive oxidative weathering processes that supply dFe with a crustal δ^56^Fe signature (Homoky et al., 2021). Nevertheless, comparison to observations reveals a few key shortcomings of this standard Fe isotope model version (Section 3.1), such as its inability to reproduce the extensive, light δ^56^Fe_diss_ anomalies associated with certain productive regions such as the Peruvian (GP16) and Mauritanian (GA03) margins, especially regarding their vertical extent. The standard experiment also produces light δ^56^Fe_diss_ features in less productive areas such as the SW Pacific or in upper parts of the NW Atlantic water column that are not observed.

Horizontal differences in sediment δ^56^Fe endmembers between margins at the same depth were better accounted for in the “C‐export sediment” experiment, where the sediment oxygenation, and consequently the sediment endmember, is modulated as a function of organic carbon export to the sediments (Section 2.4). As this carbon‐export based endmember parameterization has a lighter reductive input endmember (−2.4‰; see discussion in Section 2.4), it produces δ^56^Fe_diss_ values for the global ocean (range −1.65‰ to +3.28‰) that exceed the standard experiment's range (−0.95‰ to +3.24‰), in better agreement with observations (−3.85‰ to +2.02‰ for global data set; −1.07‰ to +1.94‰ for GEOTRACES section data set; Figure s6). In theory, this could allow the model to reproduce some of the very light values observed, for example, in the Arctic (Charette et al., 2020) or in the California Borderland basins (John et al., 2012). The light δ^56^Fe_diss_ anomalies associated with productive margins are indeed larger (and lighter) in the C‐export sediment experiment than for the standard version, with heavier δ^56^Fe_diss_ in the proximity of less productive margins (Figure 8b), leading to a better model performance for the GEOTRACES section data set and in the ocean interior (Table S1). However, the vertical extent of the light anomalies, for example, near Peruvian and Mauritanian margins, is still not well reproduced and vertical patterns of δ^56^Fe_diss_ do not always coincide with observed values. Looking globally, the C‐export sediment version appears to perform poorly at high latitudes (Figure S7), where heavy model δ^56^Fe_diss_ values are in disagreement with lighter field observations. Some of these deficiencies may be improved by also adapting the (depth‐dependent) sedimentary Fe input flux (Section 4.3), whereas others may be caused by model shortcomings such as an imbalance between ligands and dFe in the Arctic (a region that can display very light δ^56^Fe_diss_ values), or insufficient primary production (and thus carbon export) near the Mauritanian margin in our relatively coarse resolution global model (Section 4.2).

## 4 Wider Implications and Perspectives

### 4.1 Controls on Fe Isotope Cycling in the Ocean Interior

Our Fe isotope model experiments established that variable source δ^56^Fe endmembers and isotopic fractionation during certain internal cycling processes (uptake by phytoplankton and complexation by organic ligands) are both needed in the PISCES model to reproduce the observed δ^56^Fe_diss_ distribution. Generally, isotopically light dFe inputs by external sources are balanced by preferential removal of isotopically light dFe via abiotic (scavenging) and biotic (phytoplankton uptake) processes, and the partial sedimentation of this pFe pool from the water column.

The impact of internal cycling processes on δ^56^Fe_diss_ depends on the balance between abiotic removal and remineralization fluxes within the ocean interior, with the latter process dominating in the upper ocean. Abiotic removal processes mostly result in positive signatures for δ^56^Fe_diss_ as they remove light dFe, whereas remineralization often lowers δ^56^Fe_diss_ by releasing light dFe without any fractionation during the process, with notable exceptions beneath dust‐influenced or Fe‐limited surface waters. This highlights how the interplay between different Fe pools shapes not only Fe concentration but also its isotopic composition, even with only a few actively fractionating processes (Section 4.2).

The impact of hydrothermal sources on the distribution of δ^56^Fe_diss_ is evidently heterogeneous. Our model experiments suggest some Southern Ocean vents provide isotopically lighter Fe than vent systems further north, for which the large‐scale δ^56^Fe endmember associated with the effective flux may be close to a crustal composition. Sediment sources generally provide light Fe to the upper ocean and Fe with crustal endmember at depth, which reaffirms the importance of non‐reductive Fe release mechanisms operating alongside reductive supply, especially in the deep ocean. Sediment organic carbon supply, which drives reductive processes in sediments, is a better predictor for the isotope composition of Fe released from sediments in our model than the depth of the sediment‐water interface, and may therefore be used to govern both the magnitude and isotopic composition of sedimentary Fe input (Section 4.3). Dust deposition is able to affect the δ^56^Fe_diss_ in the interior via subsurface dissolution of relatively heavy Fe (compared to most other external sources) and by increasing the particle loading and thus scavenging of light Fe’. Nevertheless, its impact is largely restricted to the surface ocean, which was not a primary focus of this study (Section 4.4), and to reproduce the heavy δ^56^Fe_diss_ observations in high‐dust areas such as the North Atlantic, the combined effect of organic complexation (of heavy dFe) and scavenging (of the remaining light dFe) is required.

Finally, it is important to keep in mind that our standard Fe isotope model can only be constrained using the observational data available and may therefore underrepresent key regions and also be biased toward some uniquely regional phenomena. For instance, some transects targeted point sources in the ocean interior, such as the TAG hydrothermal vent system along the GA03 section. In addition, there can be biases linked to the geographic coverage of GEOTRACES sampling efforts to date. A key oceanic region where observations are less dense is the North Pacific, where the only field data currently available is from the SAFe station in the subtropical NE Pacific (Conway & John, 2015). At the SAFe location, observed δ^56^Fe_diss_ appear to be consistently lighter than the standard experiment output (Figure 3). This indicates that the standard parameterization does not fully capture the conditions typical of this region, such as the oxygen‐depleted zone at the SAFe site, that is widespread across the North Pacific. Consequently, the Fe isotope model may require further improvements as new observational data becomes available.

### 4.2 Interactions Between Different Fe Pools

Internal cycling emerged as a key factor in shaping the δ^56^Fe_diss_ distribution in our model experiments. Ultimately, this is a balance between fractionation during complexation, phytoplankton uptake, and multiple processes that cycle isotopes between the four pFe pools and one dFe pool in the model. Modeled δ^56^Fe_diss_ is highly variable, but within the dFe pool, due to the pre‐set fractionation, FeL is always heavier than Fe′. Phytoplankton and bulk pFe are typically lighter than the local dFe and generally have a light isotopic composition, except in Fe‐limited or high dust areas. Specifically, our results highlight the importance of overlapping processes that transfer Fe between different dissolved and particulate pools (e.g., Boyd et al., 2017), as illustrated for remineralization and abiotic removal processes through the water column in our standard experiment (Section 3.3). Factors affecting these Fe cycle processes then also modulate modeled δ^56^Fe_diss_. For instance, in the case of scavenging, the removal of light Fe′ is promoted by higher particle loads and lower ligand availability, respectively, and differences in δ^56^Fe_diss_ may indicate changes to particle and ligand concentrations if scavenging is the locally dominant process.

The importance of dFe‐pFe interactions is also evident in the real ocean, for instance, in the water column near hydrothermal and reductive sediment sources of Fe. Reducing conditions within vent fluids and sediment porewaters promote the dissolution of isotopically light Fe(II) (e.g., Homoky et al., 2009; Severmann et al., 2006, 2010), and its release to the water column. A large fraction of Fe(II) is thought to be removed close to source over short space and time scales (up to 10 s of km), for example, via precipitation of sulfides or oxyhydroxide minerals in young hydrothermal plumes (Feely, 1987) or, possibly nitrate‐dependent, oxidation and scavenging in oxygen minimum zones (Homoky et al., 2012; Lam et al., 2020; Scholz et al., 2016), processes which all may modulate its isotopic composition (e.g., Bennett et al., 2009; Chever et al., 2015; Lough et al., 2017). Over larger spatial and temporal scales, a portion of this Fe remains in solution through the formation of stable nano‐particles or Fe‐organic ligand complexes, which will each have distinct isotopic signatures (e.g., Fitzsimmons et al., 2017; Homoky et al., 2021). The largest transformation fluxes likely occur close to the input sources of Fe(II), which our global ocean model does not resolve very clearly (e.g., the very light δ^56^Fe_diss_ and dFe concentrations of around 50 μmol m^−3^ detected over the TAG hydrothermal field, Section 3.4). Consequently, for the resolution of our ocean model, we are obliged to choose sediment and hydrothermal source δ^56^Fe endmembers that integrate the primary endmember values (those which account for vent fluid/porewater processes), with any secondary transformations that occur in the water column close to the Fe input source.

Hydrothermal vents and reducing sediments have different large‐scale impacts on both dFe concentrations and δ^56^Fe_diss_ in the ocean, highlighting the influence of distinct processes in these two settings. Generally, elevated dFe concentrations from far‐field hydrothermal plumes are accompanied by an increasingly heavy δ^56^Fe_diss_ as dFe concentration decreases with distance from the local Fe input, while those linked to reductive sediments retain a light δ^56^Fe_diss_ signature. This suggests that fractionation processes operating either close to the source or during the removal and stabilization in the dispersing plume lead to a heavy hydrothermal δ^56^Fe_diss_, possibly thanks to the stabilization by organic ligands, as suggested for the SEPR plume (Fitzsimmons et al., 2016, 2017). Scavenging removal likely plays an important role in increasing plume δ^56^Fe_diss_, as it removes light dFe both in the near‐field, possibly enhanced by the presence of freshly precipitated pFe, and in the far‐field (Figure 6c). The absence of a similar fractionation in sedimentary Fe plumes indicates the supply of a more stable, light dFe species and/or particulate to dissolved transfer within the water column. Isotopically light Fe(II)‐ligand complexes may occur within surface‐sediment porewaters assuming suitable ligands are available (Klar et al., 2017). Alternatively, sediments enriched in light Fe minerals, such as those beneath OMZs, could supply light Fe, possibly stabilized by organic material (Section 4.3). It is also possible that distinct classes of ligands with different conditional stability constants may dominate in different settings, potentially introducing specific fractionation effects (Dideriksen et al., 2008; Morgan et al., 2010). Alternatively, the transfer of light Fe from pFe to dFe may lead to lighter δ^56^Fe_diss_ in the sedimentary Fe plume, as was suggested to occur on the Peruvian margin (John et al., 2018; Marsay et al., 2018), namely via remineralization (Lam et al., 2020). Indeed, in our model, remineralization releases light dFe in areas where light sediment sources dominate Fe input (Figure 6d), however, the flux is insufficient to reproduce the size of light dFe anomalies observed adjacent to the Peruvian and other productive margins beneath OMZs (Section 3.5).

More broadly, we must remember that our operational definitions of the dFe and pFe pools blend different Fe species with distinct reactivities (e.g., Tagliabue et al., 2017), and, likely, distinct isotopic composition. In particular, the emerging roles of Fe colloids need to be more directly considered in models, as they appear to be cycled distinctively compared to the “soluble” fraction of the dFe pool (von der Heyden & Roychoudhury, 2015), with at times significant differences in their respective isotopic compositions (Fitzsimmons et al., 2015). To better understand the interactions between pFe, dFe, colloids, and organic matter require specific process studies to determine the composition of each pool and the underlying processes at play. For example, it has been shown that the lithogenic‐biogenic composition of sinking pFe regulates the rate of dFe remineralization (Bressac et al., 2019). Iron isotopes may be useful in this regard, as the characteristic signatures of distinct external sources, as well as those emerging from internal transformation processes, can fingerprint different Fe pools (e.g., biogenic vs. lithogenic) and support the development of the underlying Fe cycle processes in biogeochemical models.

### 4.3 Sedimentary Fe Source Revisited

Due to the distinct δ^56^Fe endmembers associated with reductive and non‐reductive Fe input from sediments, our model reveals that the non‐reductive release of Fe with a crustal endmember widely impacts the ocean interior. This also potentially impacts upper ocean regions where lower productivity suppresses anoxia in underlying margin sediments. Moreover, we found that sediment organic carbon supply was a better proxy for the isotopic composition of sedimentary Fe input than simply using the depth of the sediment‐water interface as a proxy for sediment oxygenation and carbon demand (Section 3.5). Having here revealed the widespread significance of non‐reductive Fe inputs for the Fe isotope composition of the ocean, we also demonstrate the need to understand and improve the mass flux parameterization of the sediment source. There is evidently a need to replace the standard depth‐dependency of Fe supply in the model with a carbon‐export dependent term (e.g., Dale et al., 2015; Elrod et al., 2004), but better still, this would be augmented with a non‐reductive Fe input mechanism. However, doing so will require some careful considerations, since a simple division between reductive input of Fe(II) and non‐reductive release of Fe(III) may not be realistic without also accommodating their physical‐chemical speciation and behaviors. Added to this, factors other than carbon export will likely play a role in modulating the exchange of Fe between sediments and the water column (Homoky et al., 2016).

Models need to consider non‐reductive sediment sources, such as the postulated ubiquitous release of colloidal Fe with crustal signatures that was observed in oxic and nitrogenous sediment porewaters in the South Atlantic (Homoky et al., 2021). Consequently, factors governing such colloidal Fe release and cycling require better understanding, with key gaps related to the roles of sedimentary mineral and organic matter transformations that are able to complex Fe or stabilize Fe minerals, and the processes that facilitate the transfer of stabilized colloidal Fe to the water column. Ultimately, the influence of sedimentary Fe, delivered by different means, will also be modulated by internal cycling between Fe pools in the water column, but to different extents depending on the reactive forms supplied in the dFe pool.

For some productive and low oxygen margins such as the Peruvian margin, field observations show a broad dFe concentration signal, in both the low oxygen zones, and also at intermediate depths (John et al., 2018), in contrast to expectations from a simple depth or carbon‐export based model parameterization. This feature is thought to result from the localized release and efficient oxidative transfer of Fe(II) from shallow reducing sediments to underlying slope sediments (Scholz et al., 2014), from where Fe(III) appears to be remobilized in a more stable form allowing transport over much longer distances than for Fe(II) (Lam et al., 2020). This conceptual model is also compatible with the “rusty‐source” mechanism proposed to account for the presence of colloidal Fe with crustal isotope signature in oxidizing porewaters of the Atlantic and Southern Ocean (Homoky et al., 2021). The only difference on the Peruvian margin would be that the isotope composition of Fe(III)‐ligand or organo‐mineral complexes are not crustal, and instead bears the light δ^56^Fe signal, attributed to reductive dissolution and recycling of Fe from the shelf up‐slope. It is, therefore, possible that the release of dFe sufficiently stable for far‐field transport is highest for those sediments receiving only a moderate amount of carbon but with relatively high pFe deposition (Lam et al., 2020). If so then our carbon‐export parameterization (Equation (3a), (3b), (3c)) may require additional modifications.

While the sedimentary Fe source and its interaction with the water column may be complex and spatially diverse, a more accurate model representation is crucial, given that sediments are by far the largest source of external Fe supply in the PISCES model, and are especially important to the ocean carbon cycle (Homoky et al., 2016; Tagliabue, Aumont, et al., 2014). Future model configurations may need to consider the role of different sedimentary Fe supply vectors and how these interact with the larger‐scale ocean circulation and upper ocean nutrient limitation to modulate the ocean carbon cycle. Optimizing the sedimentary Fe source has the potential to impact modeled primary production in PISCES, both in regions where the sedimentary Fe input is currently too low, and in areas where it is overestimated and future refinements could highlight a sensitivity to other Fe sources.

### 4.4 Fe Isotope Cycling in the Upper Ocean

Fe isotopes may also help illuminate upper ocean Fe cycling, which is crucial in understanding the influence of Fe on ocean productivity. Our Fe isotope model can be used as a platform to understand the often overlapping influences of phytoplankton Fe uptake, grazing, recycling, scavenging, and bacterial dynamics that make up the “ferrous wheel” (Boyd et al., 2012; Strzepek et al., 2005). At present, our model represents the isotopic fractionation during phytoplankton Fe uptake in a simple manner and for simplicity neglects any potential fractionation by other upper ocean biogeochemical processes, such as remineralization, recycling, or bacterial Fe uptake.

Alteration to the δ^56^Fe_diss_ of the upper ocean is governed by the integrated impact of a range of fractionation processes operating simultaneously. The degree of fractionation during Fe uptake may depend on environmental conditions such as Fe availability, or could differ between species or even individual organisms, for example, due to Fe stress. Moreover, distinct Fe uptake pathways deployed by phytoplankton or bacteria, operating on distinct Fe species (e.g., Coale et al., 2019; McQuaid et al., 2018; Morrissey & Bowler, 2012) will govern the effect on δ^56^Fe_diss_. Recycling processes may actively alter upper ocean δ^56^Fe_diss_ due to Fe reduction in zooplankton guts or photochemical effects (Ellwood et al., 2015). The impact of external sources on the surface ocean may also be more intricate than presently included in our model; Fe deposition from anthropogenic sources or biomass burning carries distinct δ^56^Fe endmembers that may be locally important (Conway et al., 2019; Mead et al., 2013 Pinedo‐González et al., 2020). The impact of these different factors may exhibit significant variability on sub‐annual timescales in response to changes in Fe drawdown, phytoplankton bloom progression, shifts in the dominance of recycling, and changes in the light environment (e.g., Ellwood et al., 2015, 2020).

**Figure 9:**
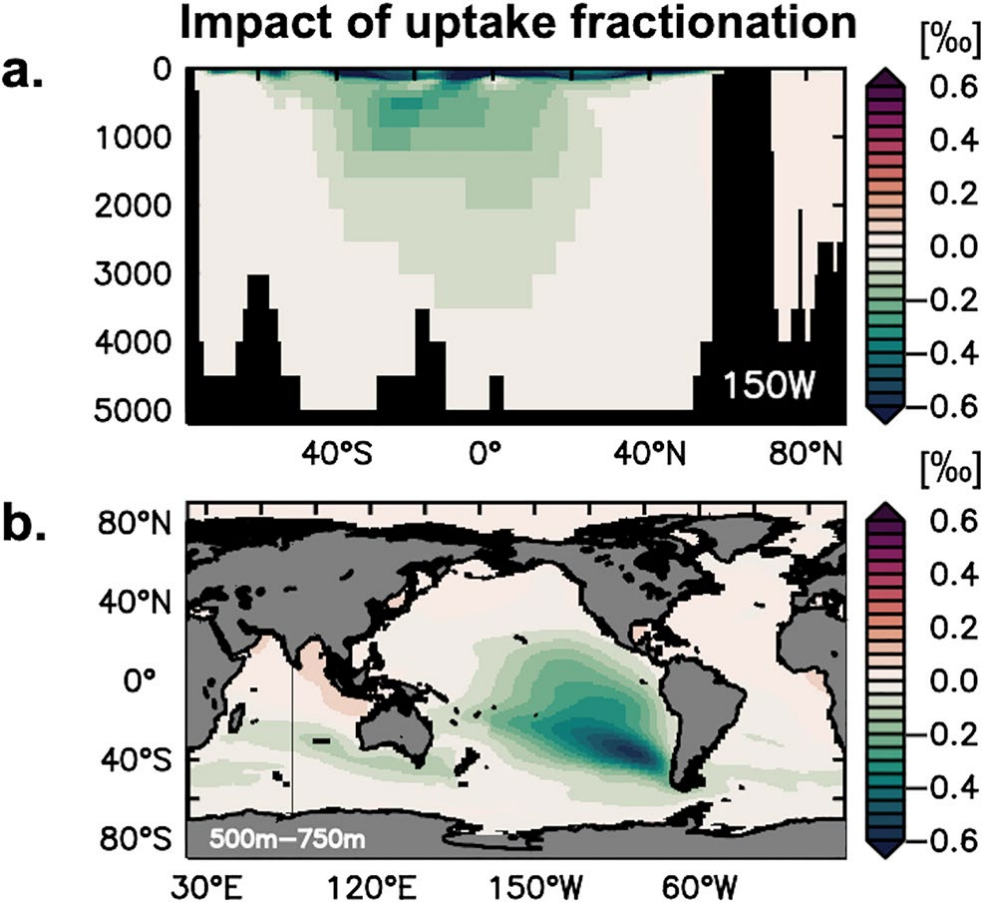
Impact of uptake fractionation on δ^56^Fe_diss_ (‰) illustrated for a meridional section in the Pacific Ocean (150°W; a) and an intermediate depth layer (500–750 m; b), calculated by subtracting the standard experiment δ^56^Fe_diss_ from that of an experiment without uptake fractionation.

Overall, these variable Fe inputs and δ^56^Fe_diss_ fractionating processes in the upper ocean indicate that Fe isotopes may be cycled on relatively small temporal and spatial scales. The signals laid down in surface waters also propagate into the ocean interior following water mass subduction at high latitudes. This can be illustrated by the change in δ^56^Fe_diss_ when biological fractionation is switched off (Figure 9). In this experiment, the impact on δ^56^Fe_diss_ is not restricted to the upper ocean, and anomalies of up to 0.5‰ propagate into the ocean interior of the Pacific from the south‐east Pacific sector of the Southern Ocean, following well‐known Antarctic intermediate water pathways. This highlights how upper ocean Fe isotope cycling, strongly mediated by biological processes, also exerts an important influence on the ocean interior, similar to that previously demonstrated for other trace metal isotopes systems such as Cd (e.g., Abouchami et al., 2014). In this regard, Fe isotopes are a useful tool. They can improve understanding of the role played by specific internal cycling processes, as well as distinguish between different algal Fe uptake mechanisms and their sensitivity to environmental change on seasonal, interannual and decadal timescales.

## 5 Conclusion

By accounting for variable source δ^56^Fe endmembers and isotopic fractionation during phytoplankton uptake and organic complexation, we were able to create a global ocean Fe isotope model that is capable of reproducing many features observed in δ^56^Fe_diss_ field data. This then allowed us to use the model as a tool to assess the role of different factors in shaping the δ^56^Fe_diss_ distribution. We find that remineralization and abiotic removal have often opposing effects on δ^56^Fe_diss_ so that the net effect depends on the relative fluxes of the two processes. Different dFe removal fluxes or transfers between dFe and pFe pools may also be responsible for the differences in large‐scale δ^56^Fe_diss_ signals associated with hydrothermal and sedimentary Fe sources, which our Fe isotope model cannot fully reproduce. Our isotope model further identifies shortcomings in the sedimentary Fe source model representation, highlighting the importance of organic matter for sedimentary Fe mobilization. Simulations also demonstrate the need to represent and improve non‐reductive sedimentary Fe input. We have gained new insight into the sources and cycling of Fe in the ocean interior by combining the latest observations with a mechanistic Fe isotope model and such approaches can contribute to better understanding upper ocean Fe sources and processes, including biological Fe cycling, in the future.

## Supporting information

Supporting Information S1

## Data Availability

Model outputs are available from https://doi.org/10.5281/zenodo.5163827 (König & Tagliabue, 2021), and field data are available as part of the Geotraces IDP and associated publications.
