# Supplementary material for: Constraints on the Cycling of Iron Isotopes From a Global Ocean Model
Source: Global Biogeochem Cycles. 2021 Sep 16;35(9):e2021GB006968. doi: 10.1029/2021GB006968 (PMC9285799; doi:10.1029/2021GB006968)
Supplement: Supplementary file 1 — Supporting Information S1 [file GBC-35-0-s001.pdf]

Global Biogeochemical Cycles

Supporting Information for

Constraints on the cycling of iron isotopes from a global ocean model

D. König[1], T. M. Conway[2], M. J. Ellwood[3], W. B. Homoky[4], and A. Tagliabue[1]

[1] School of Environmental Sciences, University of Liverpool, Liverpool, L69 3GP, UK

[2] College of Marine Science, University of South Florida, St Petersburg, FL 33701, USA

[3] Research School of Earth Sciences, Australian National University, Canberra, Australia

[4] School of Earth and Environment, University of Leeds, Leeds, LS2 9JT, UK

Contents of this file

Figures S1 to S7
Table S1

Introduction

This supporting information includes figures characterising the observational datasets used in this study (Figures S1, S6) and depicting additional information extracted from either the standard experiment (Figures S3, S5) or from model sensitivity experiments (Figures S2, S4, S7). It further includes a table depicting the performance of the standard and sediment model experiments against the GEOTRACES section dataset and against data points located at depths below 430 m (Table S1).

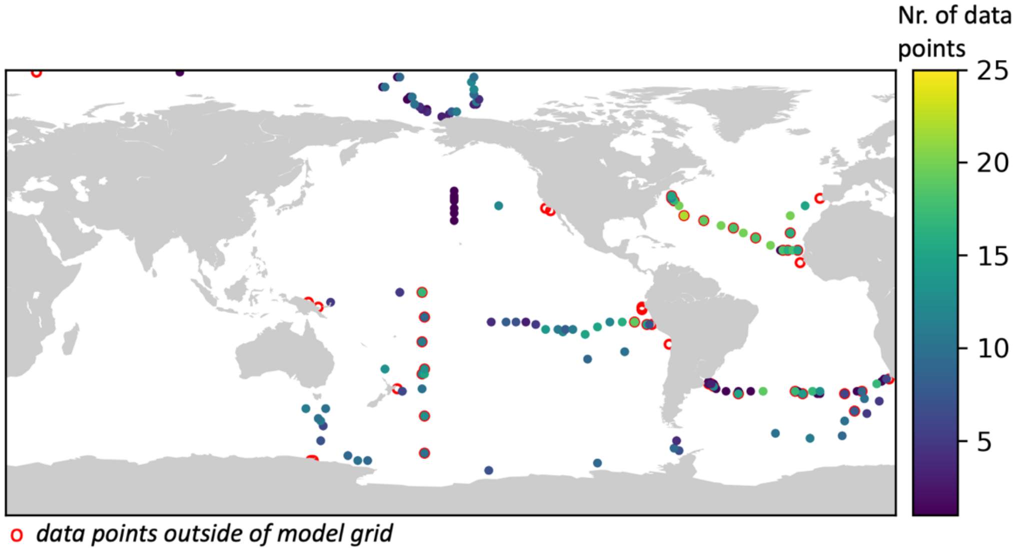

Figure S1. Observational $\delta^{56}Fe_{diss}$ data used for statistical analysis, binned into a 1° x 1° common horizontal grid and 31 model depth levels. Circles indicate locations with at minimum one data point, red circles where the location is outside the model grid, e.g., observations close to margins or below maximum model depth.

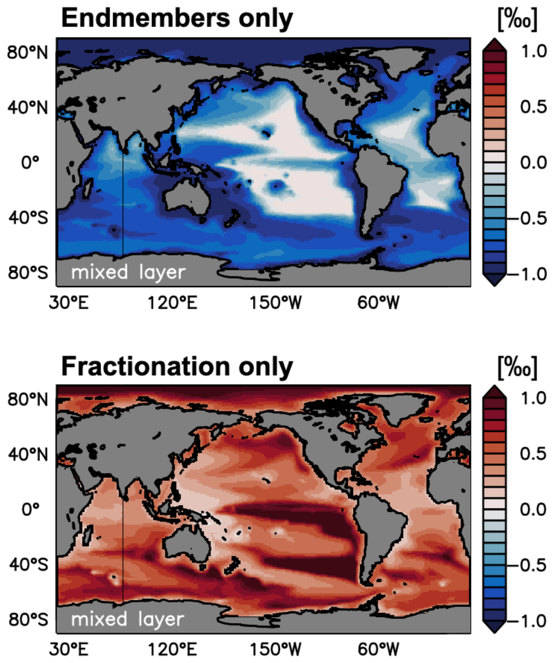

Figure S2. Average mixed layer $\delta^{56}Fe_{diss}$ (in ‰) for experiments without isotopic fractionation (a) or without source-specific $\delta^{56}Fe$ endmembers (b).

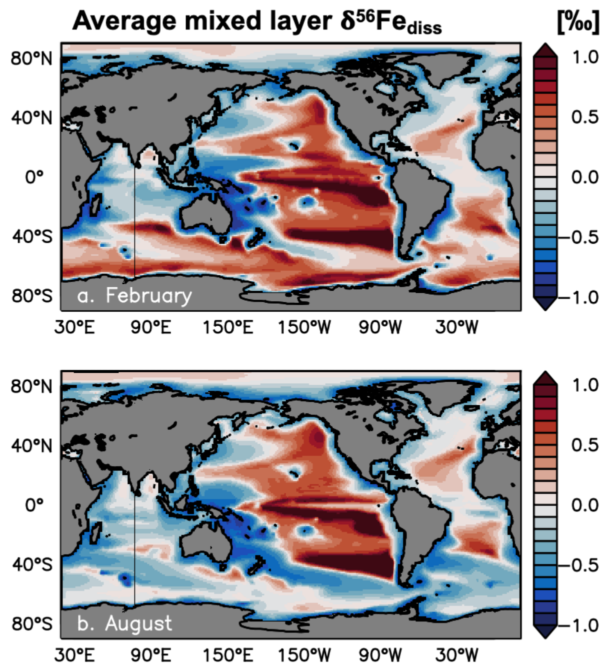

Figure S3. Monthly-averaged mixed layer $\delta^{56}Fe_{diss}$ (in ‰) for the standard experiment for February (a) and August (b), highlighting seasonal effects at mid/high latitudes (esp. the Southern Ocean).

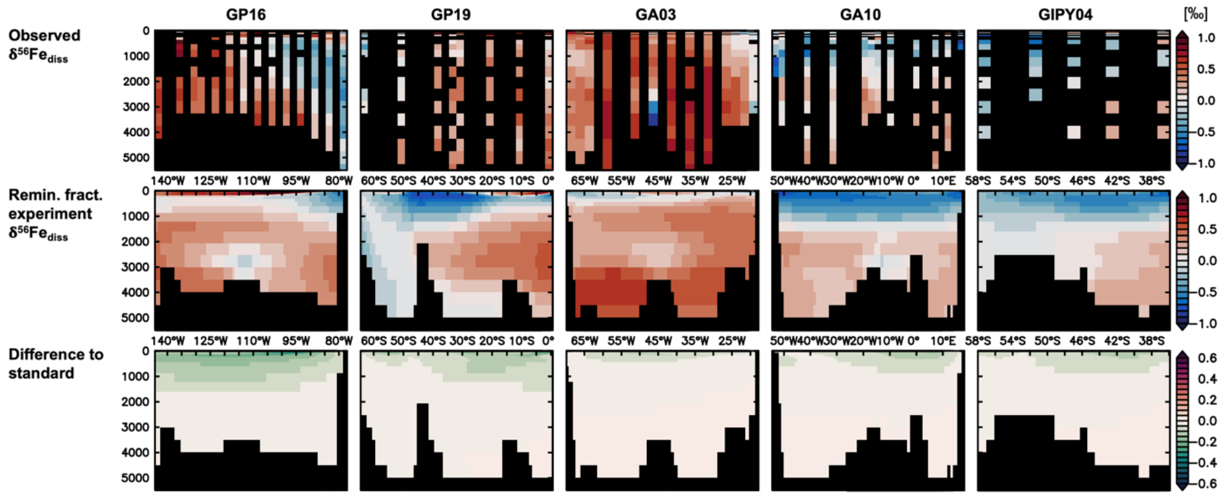

Performance against global dataset

Experiment	*Mean*	*SD*	*RMSE*	*R*	*Slope*	*Intercept*
Observations	0.12 ‰	0.52 ‰				
Remineralisation fractionation	-0.04 ‰	0.36 ‰	0.54 ‰	0.37	0.25	-0.07
Standard	0.01 ‰	0.36 ‰	0.51 ‰	0.40	0.27	-0.03

Figure S4. Comparison of the standard experiment to a version with remineralisation fractionation. The configuration of this experiment is identical to the standard experiment, except that it also includes fractionation during remineralisation of iron particles (using a fractionation factor of 0.99985) in addition to the two fractionating processes of the standard version.

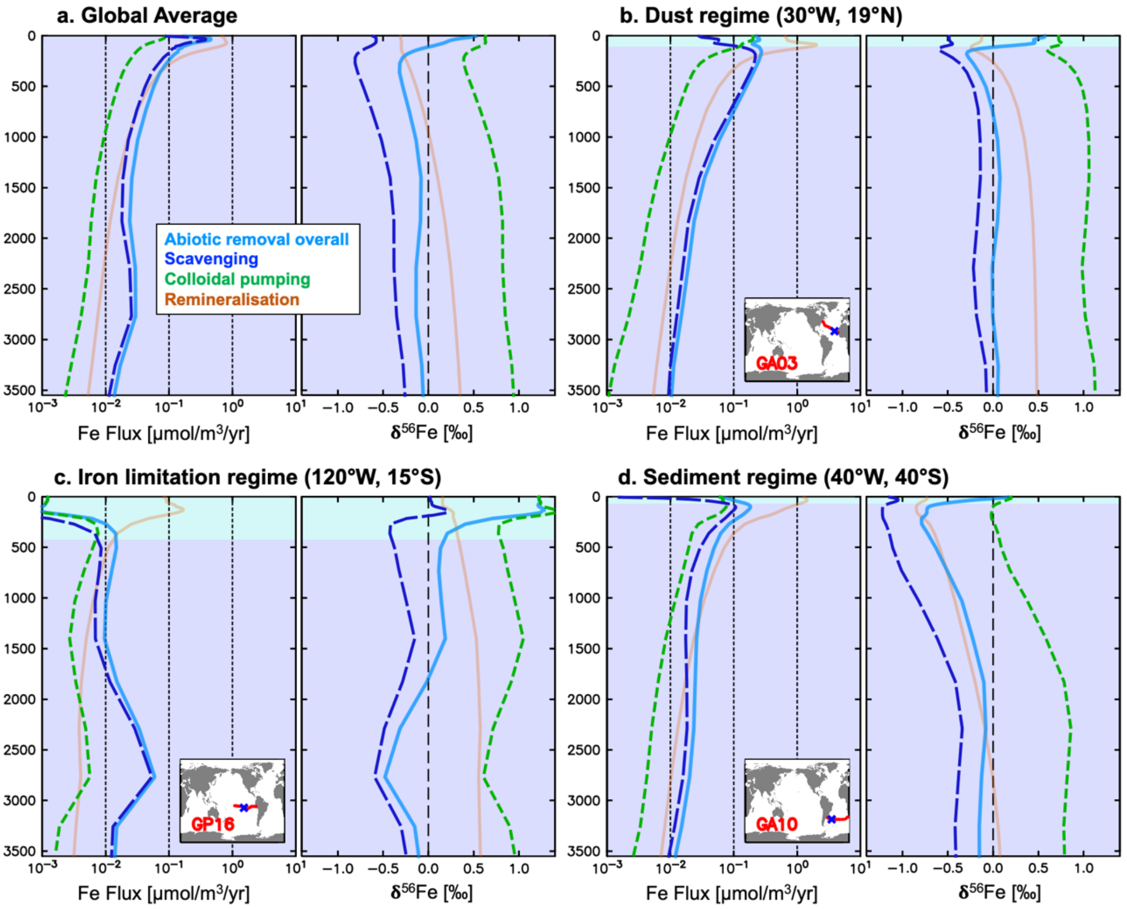

Figure S5. Depth profiles of fluxes (µmol m-3 yr-1) and δ^{56}Fe (‰) of overall abiotic removal (blue solid), and its scavenging (dark blue; dashed) and colloidal pumping (green; dashed) components. Remineralisation fluxes and signatures are shown in orange. Panels show the global average (a) and three selected locations (b-d). Background colours indicate the dominance of scavenging (dark blue) and colloidal pumping (light green), respectively.

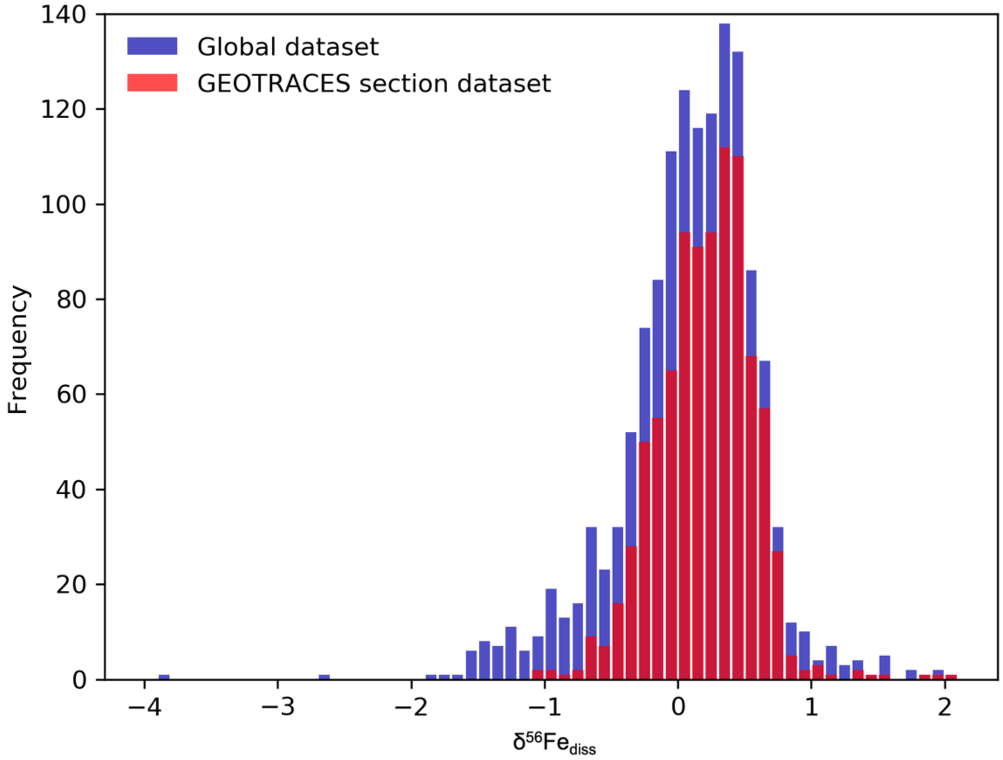

Figure S6. Histogram of observed $\delta^{56}Fe_{diss}$ for the global and the GEOTRACES section datasets.

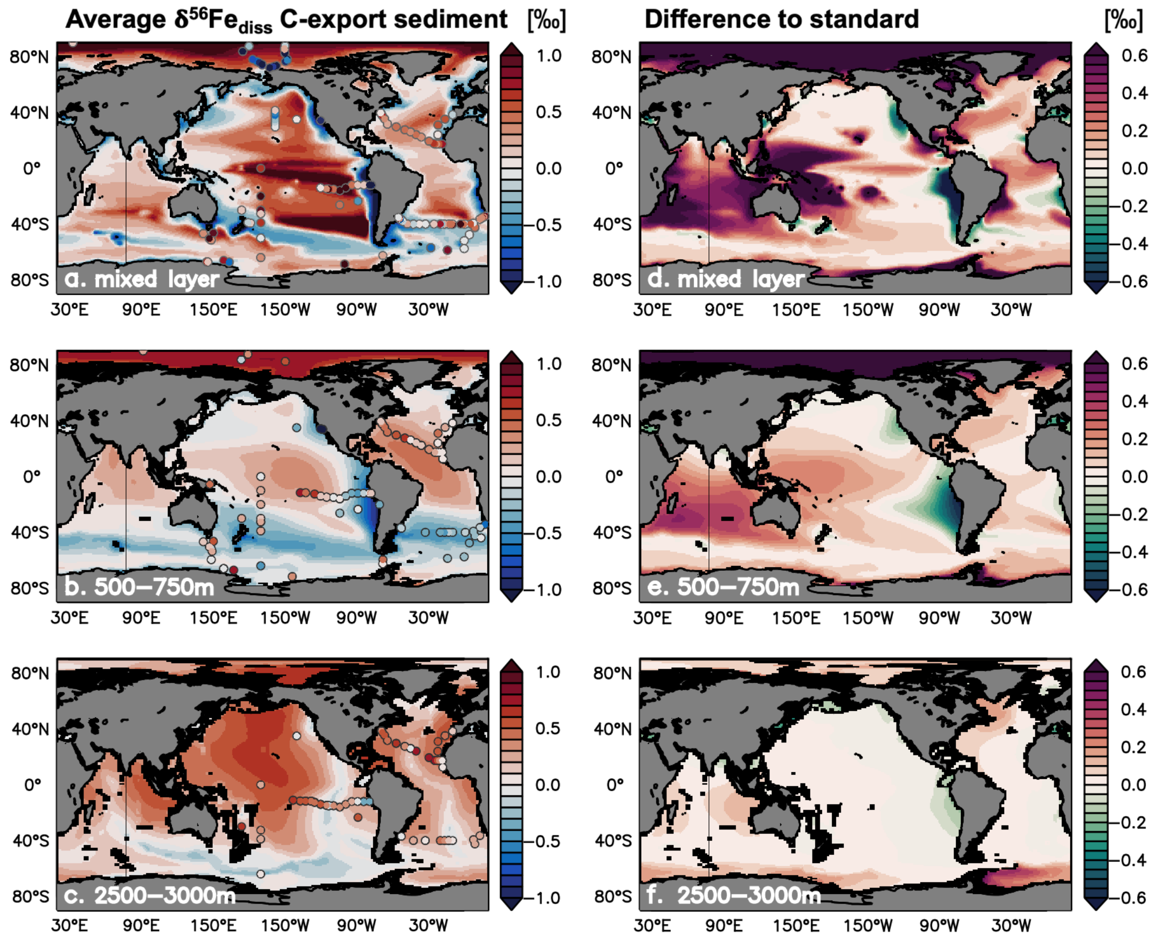

Figure S7. $\delta^{56}Fe_{diss}$ of the carbon-export dependent sediment endmember model (a-c; ‰) and its difference to the standard model (d-f; ‰), averaged over the mixed layer (a,d; average mixed layer depth), 500-750m (b,e), and 2500-3000m depth (c,f). Available observations within each depth layer are plotted on top (without averaging; a-c).

Table S1. Statistical performance of (sediment) model experiments against global and GEOTRACES section datasets, for all data points and those located below 430m

Dataset	Experiment	Nr.	Mean	SD	RMSE	R	Slope	Intercept
Global (all depths)	Observations	1197	0.12 ‰	0.52 ‰				
	Standard		0.01 ‰	0.36 ‰	0.51 ‰	0.40	0.27	-0.03
	Uniform sediment		0.06 ‰	0.31 ‰	0.51 ‰	0.33	0.19	0.03
	C-export sediment		0.15 ‰	0.41 ‰	0.65 ‰	0.03	0.03	0.15
Global (below 430 m)	Observations	510	0.21 ‰	0.34 ‰				
	Standard		0.15 ‰	0.27 ‰	0.34 ‰	0.45	0.36	0.07
	Uniform sediment		0.11 ‰	0.23 ‰	0.36 ‰	0.35	0.24	0.05
	C-export sediment		0.17 ‰	0.30 ‰	0.32 ‰	0.51	0.44	0.07
GEOTRACES section (all depths)	Observations	823	0.22 ‰	0.35 ‰				
	Standard		0.07 ‰	0.37 ‰	0.41 ‰	0.46	0.49	-0.04
	Uniform sediment		0.10 ‰	0.32 ‰	0.38 ‰	0.45	0.41	0.01
	C-export sediment		0.09 ‰	0.38 ‰	0.38 ‰	0.55	0.59	-0.04
GEOTRACES section (below 430 m)	Observations	418	0.22 ‰	0.33 ‰				
	Standard		0.19 ‰	0.26 ‰	0.28 ‰	0.59	0.46	0.09
	Uniform sediment		0.14 ‰	0.23 ‰	0.31 ‰	0.48	0.32	0.07
	C-export sediment		0.19 ‰	0.28 ‰	0.26 ‰	0.66	0.57	0.06
